# Eight habitats, 38 threats and 55 experts: Assessing ecological risk in a multi-use marine region

**DOI:** 10.1371/journal.pone.0177393

**Published:** 2017-05-10

**Authors:** Zoë A. Doubleday, Alice R. Jones, Marty R. Deveney, Tim M. Ward, Bronwyn M. Gillanders

**Affiliations:** 1 School of Biological Sciences and The Environment Institute, The University of Adelaide, Adelaide, South Australia, Australia; 2 South Australian Research and Development Institute and Marine Innovation Southern Australia, Aquatic Sciences, West Beach, South Australia, Australia; University of Sydney, AUSTRALIA

## Abstract

Identifying the relative risk human activities pose to a habitat, and the ecosystem services they provide, can guide management prioritisation and resource allocation. Using a combination of expert elicitation to assess the probable effect of a threat and existing data to assess the level of threat exposure, we conducted a risk assessment for 38 human-mediated threats to eight marine habitats (totalling 304 threat-habitat combinations) in Spencer Gulf, Australia. We developed a score-based survey to collate expert opinion and assess the relative effect of each threat to each habitat, as well as a novel and independent measure of knowledge-based uncertainty. Fifty-five experts representing multiple sectors and institutions participated in the study, with 6 to 15 survey responses per habitat (n = 81 surveys). We identified key threats specific to each habitat; overall, climate change threats received the highest risk rankings, with nutrient discharge identified as a key local-scale stressor. Invasive species and most fishing-related threats, which are commonly identified as major threats to the marine environment, were ranked as low-tier threats to Spencer Gulf, emphasising the importance of regionally-relevant assessments. Further, we identified critical knowledge gaps and quantified uncertainty scores for each risk. Our approach will facilitate prioritisation of resource allocation in a region of increasing social, economic and environmental importance, and can be applied to marine regions where empirical data are lacking.

## Introduction

Numerous anthropogenic threats are altering marine environments and the ecosystem services they provide [1–3]. Managing these threats, while balancing economic and social priorities, presents a difficult challenge for natural resource managers and policymakers, particularly in the absence of extensive empirical data. Ecological risk assessments (ERA) are used to estimate the probability of an adverse ecological effect occurring due to exposure to one or more human-mediated threats (sources of risk) and are useful for identifying and prioritising risks when data are limited. ERA frameworks can be tailored to fit the level of data available, financial and time constraints, as well as the complexity of the assessment (i.e. broad and qualitative [4, 5] versus detailed and quantitative [6]). Quantitative assessments are preferable, but are often not feasible due to the critical lack of data and resources [7, 8]. Qualitative screening-level assessments based on expert opinion are a practical first step, screening-out low risk outcomes so high risk and highly uncertain outcomes can be prioritised for more detailed evaluation.

Recognising uncertainty in qualitative risk assessments not only allows for more informed risk management, but, importantly, highlights knowledge gaps that can guide future research priorities and lead to a reduction in uncertainty [9, 10]. Knowledge-based uncertainty, associated with limited knowledge or scientific understanding [10], has previously been incorporated into opinion-based ERAs with varying levels of complexity and interpretation. Uncertainty has been considered in relation to the precautionary principle, such that higher risk scores were given by experts in the absence of adequate information [4, 11]. Similarly, risk has been determined based on the most plausible worst-case scenario, with experts providing a simple two-tier rank of uncertainty associated with that scenario [12]. Other studies incorporated an independent score-based measure of uncertainty, which was used in the overall calculation of risk [7]. The latter approach then placed greater weight on threats with higher certainty based on the premise that they have greater precision and thus greater risk. However, quantifying estimates of risk with upper and lower bounds, which are then independently retained through the risk assessment and decision-making process, can improve transparency associated with expert opinion studies, and the uncertainty surrounding that opinion. This in turn allows all options to be presented and thus better decisions to be made [10, 13].

We conducted a screening-level risk assessment encompassing multiple threats across multiple marine habitats in Spencer Gulf, Australia, a region of substantial environmental, economic and social value. Risk comprised two components, *exposure* (to the threat) and *effect* or consequence (of the exposure level), as classically-described for ERAs [13]. We aimed to develop a qualitative framework that was comprehensive (encompassing all sources of risk), flexible (suitable for a broad range of habitat and threat types), transparent and iterative, compliant with international risk management standards [14], and applicable to a range of marine systems. In brief, we used expert surveys to obtain data on the effect of a threat, a useful approach for large, screening-level assessments that incorporate multiple threats and multiple risk units (e.g. habitats, species) [7, 15], and a combination of qualitative and quantitative data (collated by authors) on threat exposure. To identify *risk range* and knowledge gaps, we also incorporated a novel measure of knowledge-based uncertainty.

## Materials and methods

This study (non-clinical) was approved by the University of Adelaide’s Human Research Ethics Committee (H-2015-234). Participants were advised that by completing the survey they consented to take part in it; a procedure approved by the Committee.

### Scope and context

**Fig 1:**
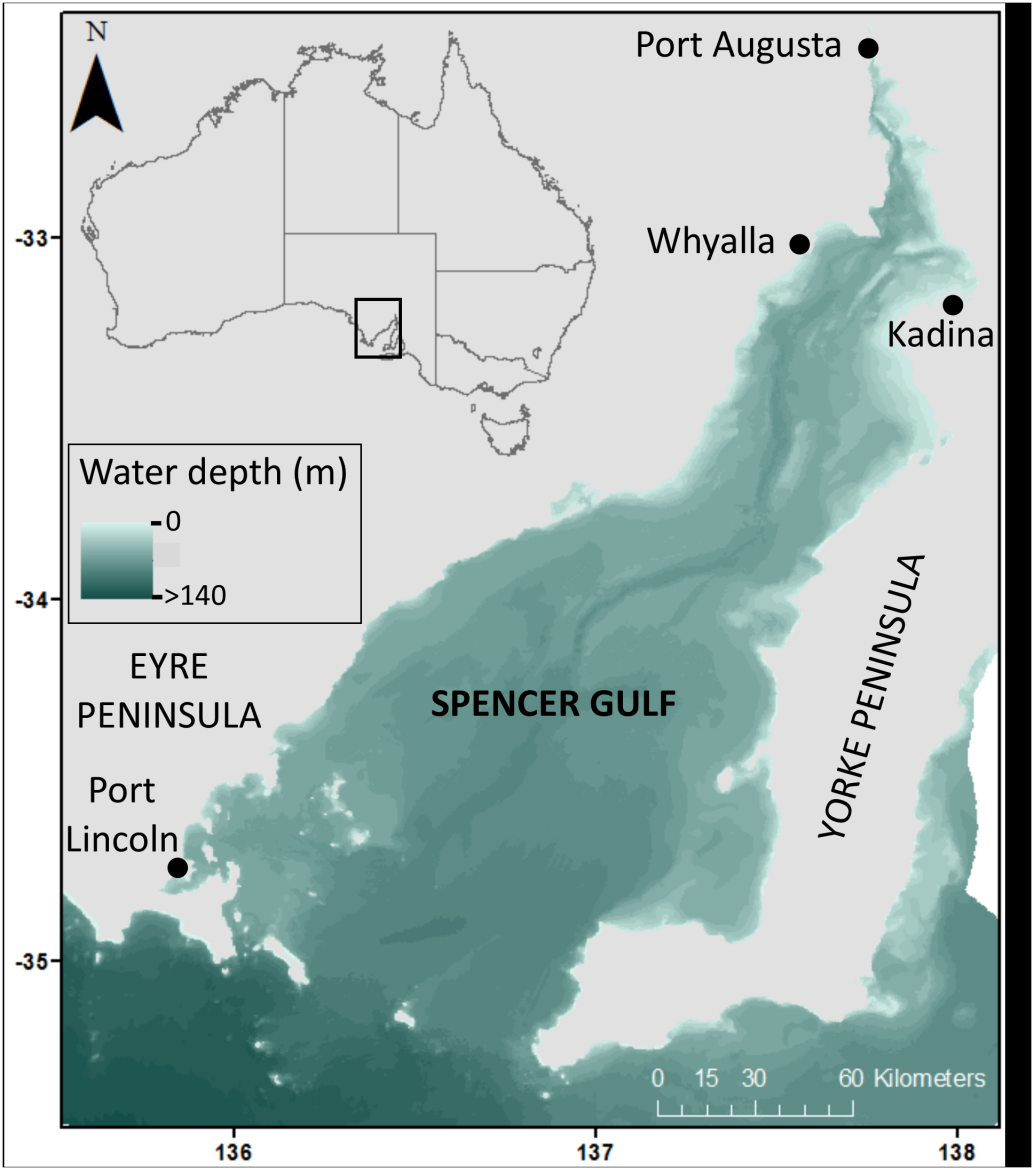
Location of study region (Spencer Gulf, South Australia).

Spencer Gulf is a large, tidal, shallow estuary spanning an area of approximately 30,000 km^2^, and is one of two gulfs within the Gulfs Province of South Australia (Fig 1) [16]. The Gulfs Province is characterised by high evaporation and low rainfall, resulting in hypersaline, inverse estuarine systems. Spencer Gulf is a relatively pristine marine region recognised for its clean, green image and high quality seafood production, as well as significant and unique environmental assets. However, it is also one of the most important economic areas in the state of South Australia and has the potential for major expansion in industrial activity, including mineral extraction and processing, shipping and port development. Like most marine regions, data on threats to Spencer Gulf are limited. As such, this risk assessment will highlight critical knowledge gaps, facilitate the prioritisation of future research and management effort, and provide a baseline for future assessments.

We defined risk as “the probability that a specified management objective is not achieved” [12], with the overarching objective to reduce negative impacts on the quality, extent and composition of marine habitats in Spencer Gulf. Eight key marine habitats (risk units; Table 1) and 38 regional and relevant global-scale threats (risk sources; see S1 Table) were identified through expert consultation and literature reviews, resulting in 304 possible threat-habitat combinations. Three other habitat types were identified in the selection process (sponge gardens, rhodolith beds and shellfish beds), however, were excluded from further assessment due to limited expertise on these habitats, and likely limited spatial coverage. The risk analysis was focussed on all potential current threats, long-term ongoing threats (climate change) and near-future (2015–2030) threats.

**Table 1:** Description of marine habitats (risk units) assessed and number of survey responses per habitat.

Habitat name	Habitat description	# surveys
Seagrasses	Intertidal and subtidal seagrasses	15
Soft bottom	Subtidal soft bottom habitats (includes sparse algal and invertebrate communities)	13
Pelagic	Pelagic habitats (all sub-tidal waters)	12
Rocky reef	Subtidal algal forest and rocky reef	11
Saltmarshes	Extratidal saltmarshes	8
Intertidal (soft)	Intertidal habitats (unvegetated soft substrate)	8
Intertidal (rocky)	Intertidal habitats (rocky substrate)	8
Mangroves	Inter- and extratidal mangroves	6

### Risk analysis

**Fig 2:**
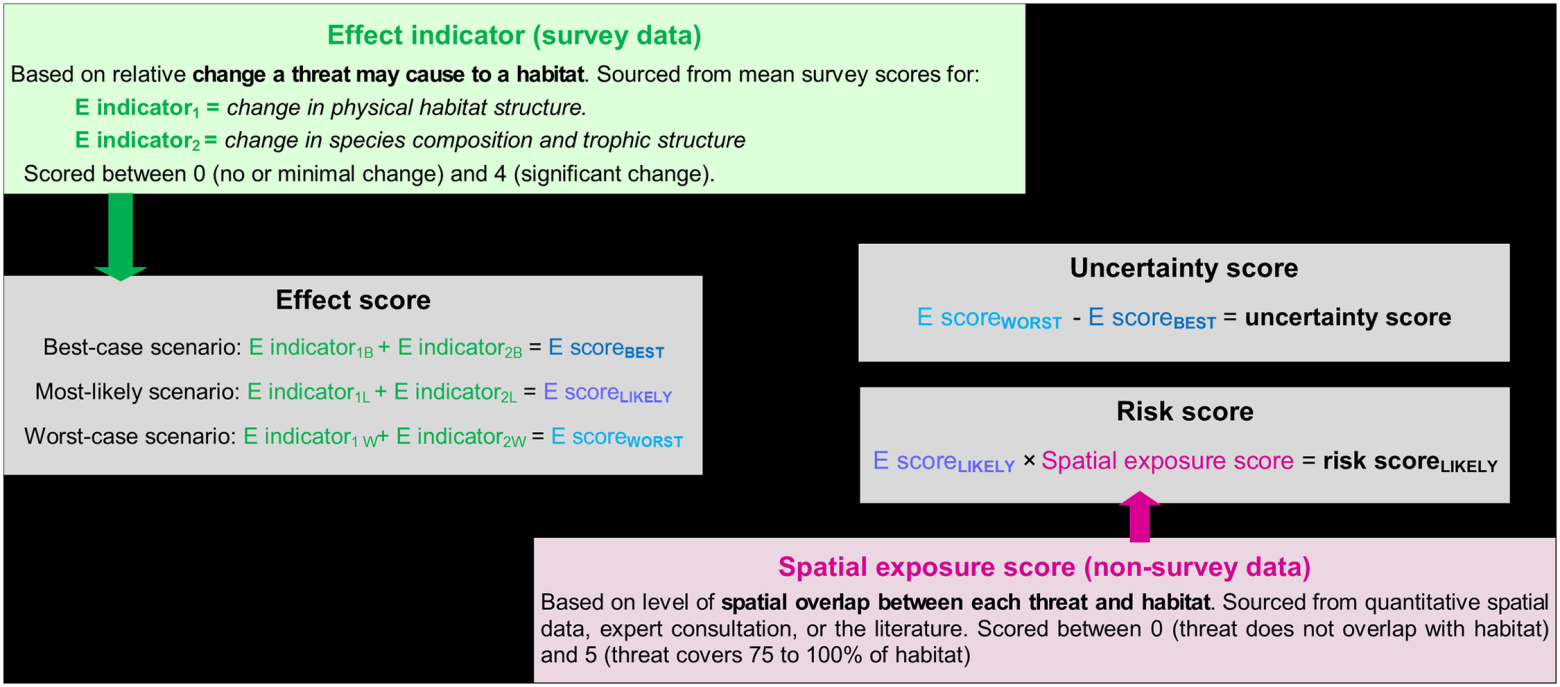
Risk assessment framework used to derive uncertainty and risk scores for each threat-habitat combination.

The risk analysis focussed on assessing the relative effect of each relevant threat to each habitat resulting from a given level of temporal exposure (i.e. the frequency at which a threat occurs in a given habitat) (S2 Table). Temporal exposure was determined through expert consultation and literature reviews and was added to each threat description in the survey. If a threat did not occur in a given habitat or where there were inadequate data to estimate exposure the threat-habitat combination was not included in the assessment. The relative effect that a single threat may cause to a specified habitat was then assessed using expert elicitation and a score-based survey and was defined using two effect indicators: 1) *change in physical habitat structure* and 2) *change in species composition and trophic structure* (Fig 2; for detailed definitions see Survey Reference Sheet in S1 File).

Experts provided three sets of scores for each effect indicator: worst-case scenario (higher scores), most-likely scenario (middle scores) and best-case scenario (lower scores) (Fig 2; see Part III in S1 File). To incorporate an independent measure of uncertainty associated with each threat-habitat effect score, and the relative ‘risk range’ based on that uncertainty, the difference between worst- and best-case scores was calculated. For instance, a high level of uncertainty or greater risk range was attributed if the two score sets were very different (e.g. 4–1 = 3) for a threat-habitat combination, whereas a smaller risk range was given if the scores were the similar or the same for each scenario (e.g. 2–2 = 0).

To obtain an overall risk ranking, the relative effect score for each threat-habitat combination and each scenario (best-case/most-likely/worst-case) was subsequently weighted according to the level of spatial overlap (%) between each threat and habitat (S3 Table). Spatial exposure scores (1 to 5) were estimated from quantitative spatial data (if available), expert consultation and literature reviews. Each spatial score was also assigned a data quality score (1 to 4), dependent on the nature of the data used to assess spatial overlap. Threats that scored a 4 (limited knowledge/no data on spatial exposure) were not included in the final risk analysis (n = 6) (S3 Table). Where quantitative spatial data on threats were available, overlap was calculated for each threat-habitat combination by overlaying a binary (presence/absence) raster grid of the threat and a map of the Spencer Gulf habitats (with ca. 250 m cell resolution). The number of cells where a habitat and threat overlapped were counted and this value was then multiplied by grid cell area before being expressed as a percentage of the total area of the specified habitat throughout the Gulf. Analyses were undertaken in R [17] using the package ‘raster’ [18] and its dependencies.

For each threat-habitat combination, a mean score for effect (i.e. from all expert responses), uncertainty and risk was calculated (Fig 2). To aid comparisons among habitats, threats and scenarios, scores were rescaled from 0 to 1. Total risk scores were also derived for each threat (i.e. the sum of all habitat specific scores) and used to create a word cloud (http://worditout.com/) with both the colour and size of the words weighted according to the total risk score.

Additional survey data were collected on perceived risk; that is, experts were asked to identify the top-five threats to Spencer Gulf without the aid of a structured framework (i.e. score-based effect indicators) (see Part III in S1 File). To rank each threat based on perceived risk, each threat listed in the top-five from each survey respondent was assigned a score from 5 (highest ranked threat) to 1 (lowest ranked threat). Scores for each threat-habitat combination were then summed and ranked, with the overall top-five threats presented per habitat.

### Survey design and distribution

We used a set of surveys, designed and distributed online using *SurveyGizmo* software, to elicit expert opinion on the ‘effect’ of each relevant threat to each habitat (S1 File). Separate surveys were created for each habitat, which were all identical apart from the list of threats in Part III (i.e. threats that did not occur in a given habitat were not included). In addition to collecting data on threat effect and uncertainty (Part III) and perceived risk (II), information was also collected on the respondents’ background and whether their expertise was related to a single bioregion in Spencer Gulf (north, central or south) (Part I). Prior to survey distribution, an example survey was assessed for readability and length by the authors and four independent researchers and was approved by the University of Adelaide’s Human Research Ethics Committee (H-2015-234). Completion time for each survey was approximately 30 minutes and questions were randomised in Part III to account for potential bias associated with survey fatigue. Over 100 experts were approached to take part in the survey. These experts were sourced from internet and literature searches, the authors’ research networks, and peer and survey respondent recommendations (passive snowballing). An expert was broadly defined as having expertise in a particular habitat(s), with *at least* a general understanding of South Australia’s gulf environments. Multiple institutions were targeted, including universities, state government agencies, and consultancies, as well as a range of experience levels (postgraduate students to retired professionals) and backgrounds (e.g. academia, natural resource management). Experts were encouraged to undertake more than one habitat survey if relevant to their expertise. The survey was open for approximately two months, with regular reminders sent to prospective respondents.

## Results

Fifty-five respondents, representing 17 institutions, completed surveys (n = 81). Most respondents were from either universities or state government agencies, and identified ‘research’ as their primary work responsibility (S4 Table). They represented a range of experience levels, in regards to both Spencer Gulf and their habitat specialty. Furthermore, there was no particular focus on a single bioregion in Spencer Gulf.

Mean effect scores (survey response values) and the variance (standard error) of the mean scores varied among threats for each habitat (S1 Fig, S1 Dataset). At the habitat level, there was a slight increase in standard error with decreasing survey sample size, with a greater level of agreement among individual survey responses for seagrass, pelagic and soft bottom habitats.

Several key differences were observed between the top-five threats based on perceived risk (Part II of survey) and the top-five threats based on risk scores. Notably, shipping and boating threats were absent from perceived risk rankings for all habitats, with greater weight placed on sediment runoff and dust (Table 2). There was also greater dominance of climate change threats associated with the risk scores. Threats with similar rankings between the two datasets included nutrient discharge, dredging and demersal trawl fishing.

**Table 2:** Top five threats for each habitat to Spencer Gulf based on perceived risk, effect scores and risk scores.

Habitat	Threats based on perceived risk	Threats based on effect scores	Threats based on risk scores
Seagrasses	P: nutrient discharge	MHM: dredging ^	CC: global warming
	P: sediment & dust	P: nutrient discharge	P: nutrient discharge
	A: fish	A: fish	CC: ocean acidification
	MHM: dredging ^	P: oil spill	Boating
	Coastal HM	MHM: ports	CC: hot weather
Soft bottom	F: demersal trawl	F: demersal trawl	F: demersal trawl
	A: fish	MHM: dredging ^	CC: acidification
	MHM: dredging ^	MHM: ports	CC: warming
	P: heavy metals	Shipping (high level) ^	Boating
	P: nutrient discharge	*P*: *nutrient discharge*	Shipping
		*P*: *oil spill*	
Pelagic	CC: hot weather events	P: oil spill	CC: ocean acidification
	CC: global warming	CC: acidification	CC: global warming
	P: nutrient discharge	Shipping (high level) ^	F: demersal trawl
	*A*: *fish*	F: demersal trawl	Boating
	*F*: *nets*	MHM: dredging ^	F: purse seine
Rocky reef	P: nutrient discharge	A: fish	CC: global warming
	P: sediment & dust	MHM: ports	CC: ocean acidification
	A: fish	P: nutrient discharge	CC: hot weather
	Coastal activities	P: oil spill	P: nutrient discharge
	CC: global warming	MHM: dredging ^	F: pots
Saltmarshes	Coastal HM	Coastal HM	CC: global warming
	CC: sea level rise *	CC: sea level rise *	CC: decreasing rainfall
	Coastal activities	MHM: marinas	CC: hot weather
	Acid sulfate soil	MHM: ports	Coastal activities
	MHM: marinas	P: oil spill	Coastal HM
Intertidal (soft)	Coastal HM	P: oil spill	*CC*: *ocean acidification*
	Coastal activities	Coastal HM	*CC*: *hot weather*
	*CC*: *sea level rise*	MHM: jetties	CC: warming
	*P*: *sediment & dust*	MHM: dredging ^	P: nutrient discharge
	*P*: *nutrient discharge*	MHM: ports	Coastal activities
Intertidal (rocky)	P: nutrient discharge	P: oil spill	CC: hot weather
	Coastal HM	Coastal HM	*CC*: *ocean acidification*
	CC: global warming	MHM: jetties	*CC*: *global warming*
	CC: hot weather	MHM: marinas	*Coastal activities*
	Coastal activities	P: nutrient discharge	*Coastal HM*
			*P*: *nutrient discharge*
Mangroves	Coastal HM	CC: sea level rise *	Acid sulfate soil
	CC: sea level rise *	Coastal HM	P: nutrient discharge
	Coastal activities	P: oil spill	CC: hot weather
	*MHM*: *marinas*	MHM: marinas	P: heavy metals
	*P*: *oil spill*	MHM: ports	P: oil spill

Each are ranked from highest (top line) to lowest (italics = equally ranked). In some instances more than five threats are listed due to equal rankings. Threats are coloured by threat category: pollution (P; orange), aquaculture (A; green) and fishing (F; green), marine habitat modification (MHM), coastal HM and activities (light blue), climate change (CC; purple), shipping and boating (grey), acid sulfate soil (cream).

Near-future threats (^) and threats lacking spatial information (*) are not included in the risk scores.

**Fig 3:**
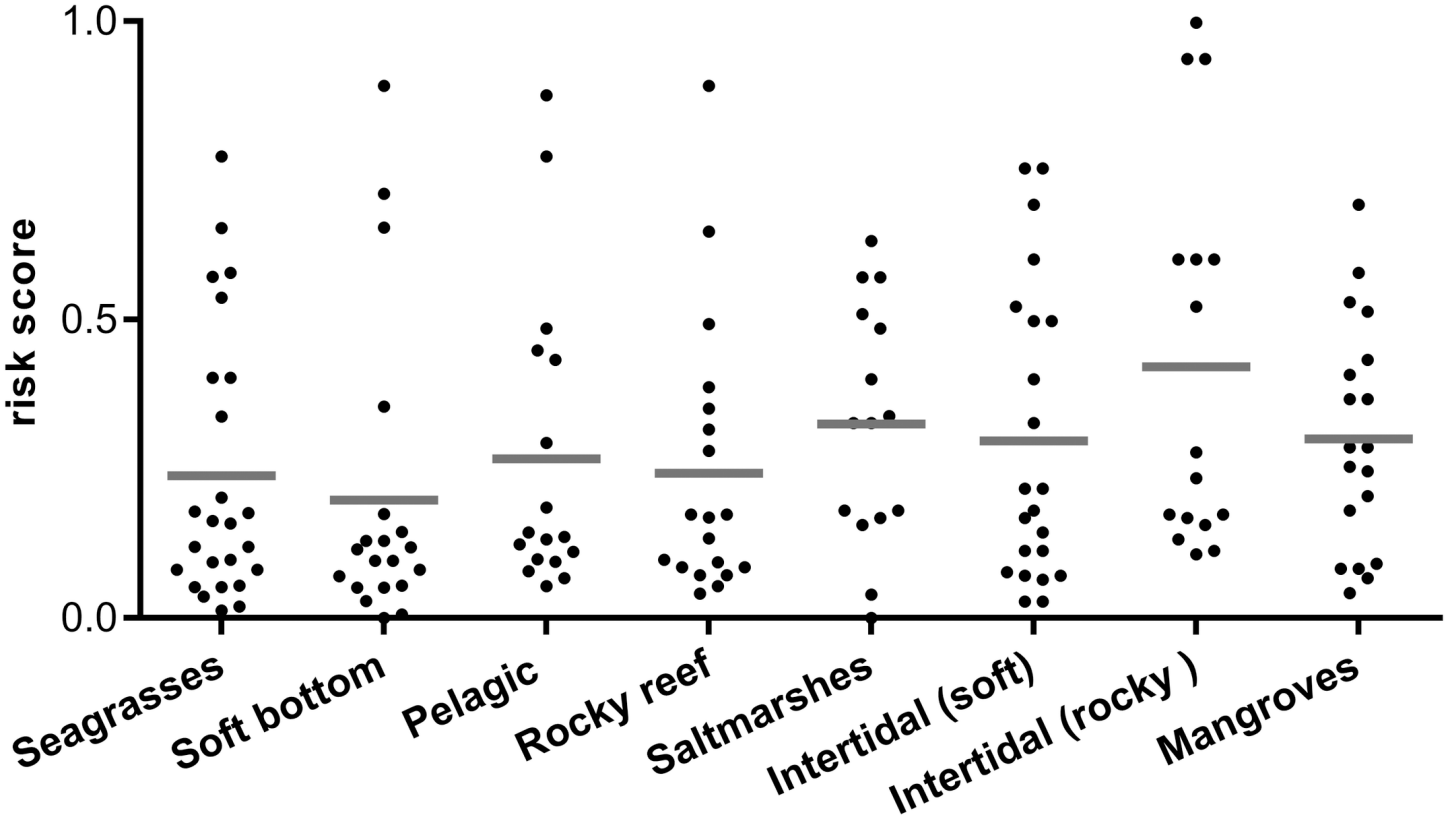
Comparison of risk scores from expert elicitation survey within and between habitats. Each point represents a risk score (most-likely scenario) for a threat-habitat combination and the grey horizontal line represents the mean of those risk scores.

**Fig 4:**
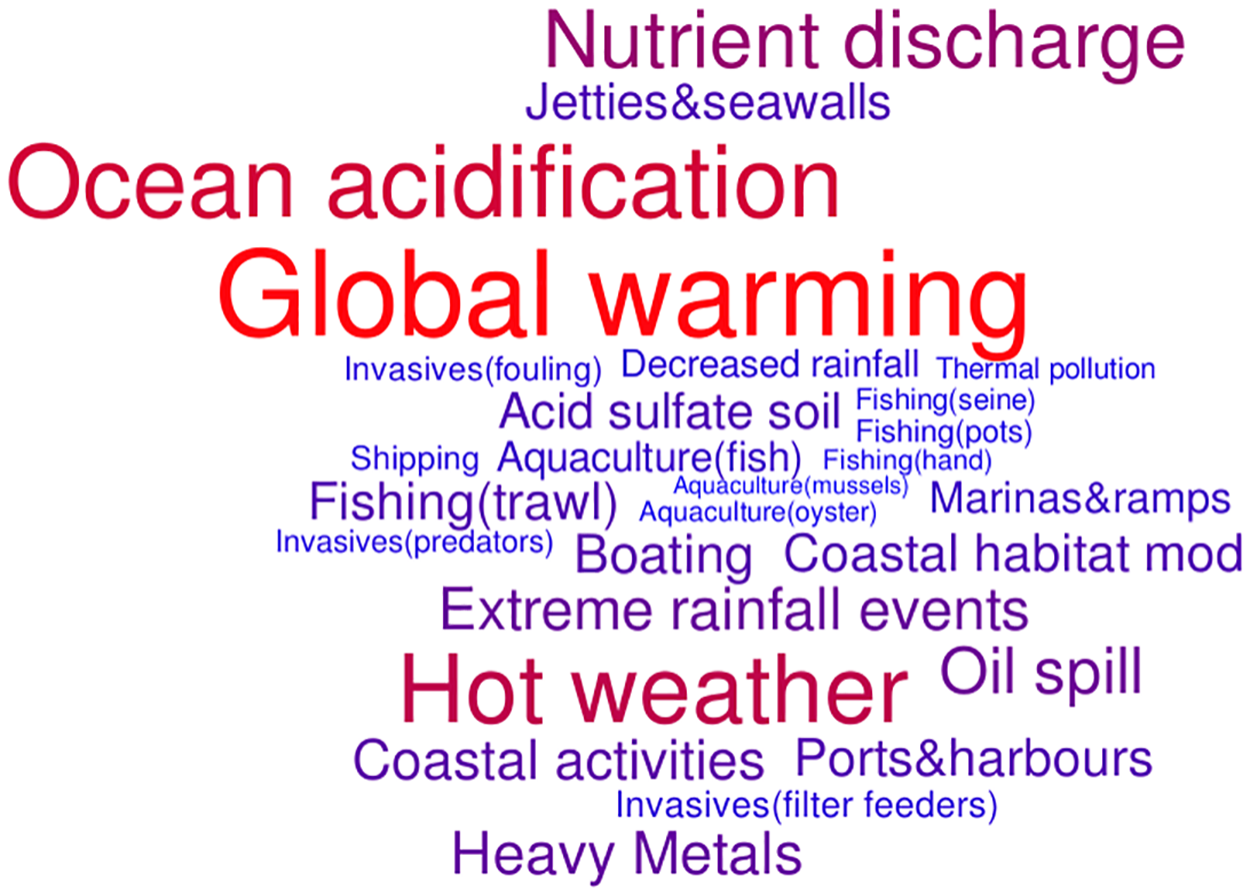
Comparison of risk level among threats, across all habitats. Size and colour of the words are weighted according to the total risk score (the sum of all habitat specific scores) for each threat (i.e. the larger and redder word = greater threat).

Subtidal habitats had a larger number of threats below the average risk level, whereas intertidal habitats had a greater number of high risk threats and an overall higher risk score (Fig 3). Overall, there was greater variation in risk among threats than among habitats. As a whole, climate change threats (specifically ocean acidification, global warming and hot weather events) were the most highly scored threats, followed by nutrient discharge (Fig 4, S2 Fig). High risk scores for climate change were influenced largely by their ubiquitous spatial coverage (i.e. high spatial exposure) across most habitats, whereas nutrient discharge had lower spatial coverage, but received very high effect scores for four habitats (Table 2). Notable high risk threats for individual habitats included boating and shipping (subtidal habitats) and acid sulfate soil (mangroves); influenced largely by high spatial exposure. Some threats with moderate spatial exposure were also ranked as high risk threats to specific habitats primarily due to their very high effect scores. These included coastal activities, habitat modification (inter-tidal habitats), and demersal trawl fishing (soft bottom and pelagic habitats). Key mid-tier threats included heavy metal pollution, extreme rainfall events, oil spills, finfish aquaculture and ports and harbours. At the other end of the spectrum, threats that were consistently ranked low risk included invasive species, shellfish aquaculture, pot (except for rocky reef habitats), line and net fishing, and thermal pollution (Fig 4, S2 Fig). Near-future threats that were not included in the final risk analysis, such as dredging and high-level shipping, received very high effect scores for five habitats (Table 2); in contrast, brine discharge from desalination plants was ranked as a mid- to low-level threat for all habitats.

Threats with high levels of uncertainty (associated with the effect of a threat) were varied and largely habitat specific (i.e. the threat of finfish aquaculture was highly uncertain for seagrass and rocky reef habitats, but not other habitats) (Table 3, S2 Fig). Data gaps exist for threats with no or very limited knowledge of spatial extent (i.e. threats that received a data quality score of 3 or 4, including sea level rise, disease outbreaks, harmful algal blooms, illegal fishing and marine debris, fishing (pot, net and line), and extreme rainfall events) (S3 Table). Even threats with a data quality score of 2 were based on limited and/or poor quality spatial data and all represent data gaps. Notably, sediment runoff and dust received very high uncertainty scores for four habitats and was also excluded from final risk calculations as spatial extent was unknown (Table 3). Many threats that received a high uncertainty score or were not included in the risk analysis due to limited spatial data, also had high risk and/or effect scores, including many climate change threats, pollution (inputs from finfish aquaculture, nutrient discharge, heavy metals, and oil spill) and habitat modification (marine and coastal). Knowledge gaps also exist for the benthic habitats that were not included in this risk analysis (sponge gardens, rhodolith beds and shellfish beds), due to the absence of even qualitative data.

**Table 3:** Top-five threats for each habitat, ranked by the highest uncertainty scores (*). ‘Y’ indicates whether a threat-habitat combination also received a “top five” effect and/or risk score (see Table 2).

Threat	1	2	3	4	5	6	7	8	Top-5 CS	Top-5 RS
Aquaculture: fish	*			*					Y	
Acid sulfate soil					*					Y
Climate change: decreasing rainfall					*					
Climate change: global warming			*			*				Y
Climate change: hot weather	*									Y
Climate change: ocean acidification		*		*						Y
Climate change: sea level rise								*	Y	NA
Coastal activities					*					Y
Coastal habitat modification					*		*	*	Y	Y
Disease outbreaks							*			
Fishing: demersal trawl	*								Y	Y
Harmful algal blooms			*							
Invasive species: encrusting, fouling				*						
Invasive species: filter feeders		*				*	*			
Marine habitat modification: dredging		*			*				Y	NA
Marine habitat modification: jetties, seawalls							*		Y	
Marine habitat modification: marinas, boat ramps	*				*				Y	
Marine habitat modification: ports, harbours	*				*			*	Y	
Pollution: brine							*			NA
Pollution: heavy metals			*			*		*		Y
Pollution: nutrient discharge			*	*					Y	Y
Pollution: oil spill		*	*	*	*	*			Y	Y
Pollution: sediment & dust		*				*	*	*		NA

In some instances more than 5 threats are listed per habitat due to equal rankings. Table cells are coloured according to the quality of the spatial data: adequate spatial data (yellow), limited spatial data or well-documented ‘whole-of-habitat’ threat (orange), limited knowledge/no spatial data (red). Near-future threats are not coloured, as there are no spatial data available for these. NA = not applicable, risk scores not calculated because of a lack of spatial data. 1 = seagrasses, 2 = soft bottom, 3 = pelagic, 4 = rocky reef, 5 = saltmarshes, 6 = intertidal (soft), 7 = intertidal (rocky), 8 = mangroves.

## Discussion

Our assessment identified key threats to eight habitats from 304 threat-habitat combinations to guide management and research prioritisation and resource allocation in Spencer Gulf. Pervasive, ongoing threats associated with climate change (e.g. global warming, ocean acidification and hot weather events) were consistently ranked as high risk (Fig 4). Comparable studies, indicating the relative impact different threats pose to habitats or relative vulnerability of habitats to multiple threats, also identified climate change as a high risk threat. Although only broad comparisons can be made due to varying methods, scales and threat-habitat categories, global warming, ocean acidification, sea level rise, and, to a lesser extent, increase in ultraviolet radiation, were consistently identified as major threats to the Californian Current [19], Northwestern Hawaiian Islands [20] and Mediterranean and Black seas [21], as well as globally [1, 7]. Even though climate threats cannot be managed directly at the regional scale, identifying these threats at the screening-level is important, because management of regionally-specific threats may ameliorate or exacerbate the effects of climate change [22, 23]. There is also a general consensus among habitat-threat studies that small, intertidal habitats are at greater risk than their larger subtidal counterparts due to exposure to high-impact, land-based human activities, such as coastal development and habitat modification (e.g. this study [19, 20, 24, 25]). Similarly, demersal fishing is commonly identified as a high risk threat to marine habitats [19, 21, 24], and was the highest-ranked fishing-related threat to Spencer Gulf. Shipping and boating related threats were also key threats to some habitats in Spencer Gulf, as in other marine regions [20, 21], and may become a greater threat to the region if shipping and associated dredging and port development increase as predicted [26]. Interestingly, shipping and boating threats, which receive comparatively less research and media attention than many of the other threats, were absent from the perceived risk rankings (i.e. what experts thought were high risk threats prior to undertaking the survey; Table 2).

Comparisons among studies also highlights that the level of risk different threats pose to marine habitats are generally context specific and need to be interpreted as a measure of relative risk within a system. Nutrient pollution, for example, was the highest ranked, regional-scale stressor to Spencer Gulf, which is not surprising given that it is an oligotrophic environment and low-level nutrient discharge could cause significant change. In contrast, on a global scale, nutrient pollution was ranked as a relatively low tier threat to comparable habitats [7]. In this study, invasive species, and most of the fishing-related threats, were consistently ranked as low risk across all relevant habitats, whereas in many areas these are key threats to the marine environment, and have been identified as such in regional and global scale assessments [1, 19, 24, 25]. This result is not surprising in the regional context; Australian fisheries are well managed relative to many other countries [27]. In Spencer Gulf, furthermore, the ecological footprint of invasive species is poorly documented and therefore often identified to be relatively minor, and the hazards and associated risk profiles poorly understood [28] in comparison to other regions [29]. Further, locally-relevant threats, which can otherwise be overlooked in broader-scale assessments, may pose a significant risk to local habitats, such as acid sulfate soil disturbance to mangroves in Spencer Gulf and invasive species to coral atolls in the Hawaiian Islands [20]. This emphasises the importance of undertaking regional assessments using local expertise and data layers and considering threats applicable to the region.

Our study highlighted important knowledge gaps through each stage of the assessment, from lack of experts for known habitats in Spencer Gulf (e.g. shellfish beds) to limited data on threat exposure (e.g. marine debris, harmful algal blooms) and to a quantifiable measure of knowledge-based uncertainty (i.e. a risk ranking with upper and lower bounds) associated with each threat effect (e.g. Table 3). Knowledge-based uncertainty, if considered at all in ecological risk assessments, has been incorporated and defined in various and sometimes opposing ways [7, 11, 12]. Our novel approach involved asking experts to score the best, most-likely and worst-case effect of each threat to each habitat, which were then independently retained in the assessment (see S2 Fig), rather than merged into a single uncertainty-risk value with the assessors’ interpretation of uncertainty applied. This independent measure of knowledge uncertainty allows for more transparent and informed decision making and can direct research planning so that uncertainty, particularly associated with highly ranked threats (e.g. climate change), can be reduced. We acknowledge that expert estimates are influenced by other sources of uncertainty, such as linguistic uncertainty (interpretation of terms), natural ecological variation, human error, and social-cultural precepts that shape the perception of risk [9, 10], which are not incorporated here. Although all quantifiable sources of uncertainty should be acknowledged where feasible, we also emphasise that screening-level risk assessments are a starting point to investigate high risk and highly uncertain threats in more detail.

In addition to incorporating all quantifiable sources of uncertainty, we recommend that future detailed risk assessments should encompass two important additional components. First, as multiple threats are present at any one time, interactive effects among threats are likely [30]. As such, antagonistic or synergistic interactions between key threats should be considered, even if qualitative data are only available. This would be particularly relevant for climate change threats, whereby a reduction in a regionally managed threat, such as nutrient input, could counteract the impact of global climate change if a synergistic interaction were present [22, 23]. Second, a future risk assessment should incorporate social and economic risks, which are intrinsically linked to ecological risk. Social-ecological assessments may involve assessing how ecological risk may effect ecosystem services provided by marine habitats (e.g. provision of food, regulation of the environment, and cultural benefits), the value that stakeholders place on these services, and how much the social-ecological systems can adapt to changes in these services (i.e. adaptive capacity) [31–33]. For such assessments, qualitative data would be obtained from a broader cross section of the community, including recreational users, indigenous groups, NGOs and local industry and government agencies.

## Conclusions

Marine science is largely under-resourced [8] and regional-scale ecological risk assessments can provide critical guidance for research and management priorities. Our assessment identified key threats to a high-value, multi-use marine region from 304 threat-habitat combinations. This was achieved with limited resources and empirical data by using expert opinion and survey-based elicitation methods. Key principles underlying international risk management principles are transparency and the use of a framework that is iterative and flexible [14]. Screening-level risk assessments based on quantifiable survey data can be repeated in time to determine if risk rankings have changed due to management inventions or the acquisition of new qualitative or empirical data (which could alter a threat’s risk rating significantly), or can be used as a springboard to undertake detailed holistic assessments on high risk threats. The framework presented here can be applied to marine regions where empirical data are lacking, and, importantly, provide a unique set of data on knowledge gaps and uncertainty associated with each habitat-specific risk ranking. These data could be used in a cumulative impact mapping study [1, 21], whereby both the cumulative risk of multiple threats and the cumulative uncertainty associated with that risk could be mapped independently.

## Supporting information

S1 Table Description of all threats (sources of risk) used in the risk analysis (n = 37). All threats are either current threats, long-term ongoing threats^#^ (climate change) or near-future threats* (2015 to 2030). Information concerning climate change threats (i.e. past and near-future 2030 predictions) were sourced from the Australian Bureau of Meteorology. HM = Habitat modification.
(DOCX)

S2 Table Average frequency (temporal exposure) for each threat and each habitat in Spencer Gulf. 0 = threat does not overlap with habitat, 1 = threat is a rare event (less than once a year), 2 = threat occurs 1 to 30 days/year, 3 = threat occurs 1 to 3 months/year, 4 = threat occurs 3 to 9 months/year, 5 = threat is near-continuous to continuous. HM = habitat modification, * = specific frequency based on climate change predictions (60 days/year).
(DOCX)

S3 Table Spatial exposure scores for each threat-habitat combination in Spencer Gulf. 0 = threat does not overlap with habitat, 1 = threat covers < 10% of habitat, 2 = threat covers 10 to 25% of habitat, 3 = threat covers 25 to 50% of habitat, 4 = threat covers 50 to 75% of habitat, 5 = threat covers 75 to 100% of habitat, dash = no available data. HM = habitat modification. Data quality category = adequate spatial data (1), limited spatial data or well-documented ‘whole-of-habitat’ threat (2), expert opinion/qualitative data (3), limited knowledge/no data (4), not applicable, near-future threat (NA).
(DOCX)

S4 Table Background information associated with survey respondents (n = 55). Statistics below are based on number of surveys and percentage of the total number (n = 81).
(DOCX)

S1 Fig Level of variance in survey response values (raw effect scores, most-likely scenario) among groups (a; mean) and within groups (b; standard error) for each habitat, with habitats ordered from highest sample size (seagrass; n = 15) to lowest (mangroves; n = 6). Each point represents the mean or standard error of the effect score for each threat-habitat combination and the grey horizontal line represents the grand mean of those points.
(DOCX)

S2 Fig Risk assessment result for each habitat. *Left-hand graph*: effect scores for each threat and scenario. Scenario effect scores are indicated by vertical black dashes, ranging from best-case (yellow), most-likely and worst-case (red). Uncertainty scores (effect score_worst_ minus effect score_best_) for each threat are indicated by crosses. Threats are ranked from the highest to lowest most-likely effect. Near-future threats (^) and threats lacking spatial information (*) are not included in the final risk analysis. *Right-hand graph*: risk scores for each threat based on most-likely scenario, ranked from highest to lowest risk. Numbers relate to data quality category of the spatial information: adequate spatial data (1), limited spatial data or well-documented ‘whole-of-habitat’ threat (2), expert opinion/qualitative data (3), limited knowledge/no data (4).
(DOCX)

S1 File Example survey. Includes Participant Information Sheet and Survey Reference Sheet.
(DOCX)

S1 Dataset Raw data (individual survey response scores). (XLSX)
