# Supplementary material for: Eight habitats, 38 threats and 55 experts: Assessing ecological risk in a multi-use marine region
Source: PLoS One. 2017 May 10;12(5):e0177393. doi: 10.1371/journal.pone.0177393 (PMC5425208; doi:10.1371/journal.pone.0177393)
Supplement: S1 Table — All threats are either current threats, long-term ongoing threats# (climate change) or near-future threats* (2015 to 2030). Information concerning climate change threats (i.e. past and near-future 2030 predictions) were sourced from the Australian Bureau of Meteorology. HM = Habitat modification. (DOCX) [file pone.0177393.s001.docx]

**Table S1.** Description of all threats (sources of risk) used in the risk analysis (n = 37). All threats are either current threats, long-term ongoing threats^#^ (climate change) or near-future threats* (2015 to 2030). Information concerning climate change threats (i.e. past and near-future 2030 predictions) were sourced from the Australian Bureau of Meteorology. HM = Habitat modification

| **Threat** | **Description** |
| --- | --- |
| Acid sulphate soil disturbance | - Also includes acid water drainage from acid sulphate soil disturbance |
| Aquaculture: mussels | - Growout, sub-tidal sub-surface longlines |
| Aquaculture: Pacific oyster | - Growout, intertidal rack-and-rail and longlines - Sub-threats include trampling, sedimentation, shading with rack-and-rail - Does not include impacts from invasive feral populations or hatcheries |
| Aquaculture: predatory fish (plus diffuse nutrients) | - Growout, sea cages, southern bluefin tuna and yellowtail kingfish - Main threat includes high diffuse nutrient input - Does not include impacts from hatcheries or introduced pathogens |
| Boating | - From recreational activities, commercial fishing, aquaculture operations and ecotourism - Sub-threats include grounding, sediment re-suspension, anchor damage, waste discharge and antifoulants - Does not include threats related to invasive species or fishing |
| Brine discharge* | - From a desalination plant - While no desalination plants currently exist in spencer gulf, it is expected that they will be developed in the near-future |
| Climate change: ocean acidification^#^ | - Decrease in ocean pH by 0.07 - Consider that ocean pH has already decreased over the past 200 years by about 0.1 |
| Climate change: global warming^#^ | **For inter-tidal and sub-tidal habitats:**   - Increase in mean sea surface temperature by 0.8°C - Consider that SST increase, over the past 100 years, has been relatively minimal for the South Australian region (< 0.1°C)   **For inter-tidal and extra-tidal habitats:**   - Increase in mean air temperature by 0.4 to 1.1°C - Consider that air temperature, over the past 100 years, has already increased by around 0.7°C in the South Australian region |
| Climate change: sea level rise^#^ | - Rise in mean sea level by 120 mm (12 cm) - Consider that sea level has been rising along the Australian coastline about 2.1 mm/year over the past 50 years - Sub-threat includes increases in extreme sea level events (e.g. Astronomical tides, storm surges and wind waves) |
| Climate change: increase in hot weather events^#^ | - Increase in number of hot days (air temperature > 35°C) to 60 days/year - Consider that most of the Spencer Gulf region has experienced about 30 hot days/year over the past 30 years |
| Climate change: increase in extreme rainfall events^#^ | - Increase in the intensity, rather than frequency, of rainfall events. Magnitude of increase unknown. |
| Climate change: decrease in rainfall^#^ | - 15% decrease in winter rainfall - Consider that there has been a prolonged period of drying in the South Australian region since the 1990s |
| Coastal activities | - Such as walking, sunbathing, off-road vehicles and grazing |
| Coastal habitat modification | - Such as land reclamation, native vegetation removal and shoreline hardening - Does not include threats associated with increased coastal activities, sediment runoff and acid sulphate soil disturbance |
| Disease & pathogen outbreaks | - Outbreaks from naturally occurring and introduced species - Does not include harmful algal blooms |
| Fishing: hand collection | - Abalone, commercial and recreational |
| Fishing: handline, longline | - Scalefish, commercial and recreational - Sub-threats include low level bycatch |
| Fishing: haul nets, gillnets | - Scalefish, commercial and recreational - Sub-threats include medium level bycatch |
| Fishing: pots | - Blue crab and rock lobster, commercial and recreational - Sub-threats include low level bycatch |
| Fishing: demersal trawl | - Western king prawns, commercial - Sub-threats include high level bycatch and sediment re-suspension |
| Fishing: purse seine | - Sardines, commercial - Sub-threats include low level bycatch |
| Fishing: illegal | - All types |
| Harmful algal blooms | - Outbreaks from naturally occurring and introduced species |
| Heavy metals | - Point source and diffuse pollution from historical mining operations, stormwater, metal manufacture and power stations |
| Invasive species: benthic filter-feeders | - Key species include European fanworm, Pacific oyster and pearl oyster |
| Invasive species: encrusting, fouling | - Key species include ascidians, hydroids and macroalgae |
| Invasive species: predators, parasites | - Key species include crabs, goby fish and shell-boring worms |
| Marine debris | - Includes general litter, fishing gear, aquaculture gear and illegal dumping |
| Marine HM: commercial harbors, ports | - Sub-threats include alteration of currents/tides, sedimentation and coastal erosion |
| Marine HM: jetties, seawalls | - Sub-threats include alteration of currents/tides, sedimentation and coastal erosion |
| Marine HM: marinas, boat ramps | - Sub-threats include alteration of currents/tides, sedimentation and coastal erosion |
| Marine HM: dredging* | - Sub-threats include alteration of currents/tides and sediment re-suspension |
| Nutrient discharge (point source) | - Mainly nitrogen and phosphorous - Point-source pollution from wastewater treatment plants, stormwater drains, steel manufacture, fish processors, power plants, land-based abalone farms, aquaculture (finfish) hatcheries, agricultural runoff via riverine/creek discharge and shark-cage berley - Does not include impacts from sea cage aquaculture |
| Oil spill | - The most-likely, worst-case oil spill for Spencer Gulf. For example, the 1992 *‘ERA’* spill: fuel source = ship’s fuel from ruptured fuel tank; fuel type = highly-persistent heavy fuel oil not easily broken down by chemical dispersants; spill size = 100s of tonnes. |
| Sediment runoff & dust | - From land clearance and coastal habitat modification |
| Shipping | - > 100 ships crossing a habitat per year - Sub-threats include sediment re-suspension, anchor damage, waste discharge, noise and antifoulants - Does not include threats related to invasive species or oil spills |
| Shipping (high level)* | - As above, but based on near-future predictions for shipping intensity in Spencer Gulf (300 to 600 ships crossings per year) |
| Thermal pollution | - Point-source from power plants and steel manufacture |
