# Supplementary material for: Eight habitats, 38 threats and 55 experts: Assessing ecological risk in a multi-use marine region
Source: PLoS One. 2017 May 10;12(5):e0177393. doi: 10.1371/journal.pone.0177393 (PMC5425208; doi:10.1371/journal.pone.0177393)
Supplement: S2 Table — 0 = threat does not overlap with habitat, 1 = threat is a rare event (less than once a year), 2 = threat occurs 1 to 30 days/year, 3 = threat occurs 1 to 3 months/year, 4 = threat occurs 3 to 9 months/year, 5 = threat is near-continuous to continuous. HM = habitat modification, * = specific frequency based on climate change predictions (60 days/year). (DOCX) [file pone.0177393.s002.docx]

**Table S2.** Average frequency (temporal exposure) for each threat and each habitat in Spencer Gulf. 0 = threat does not overlap with habitat, 1 = threat is a rare event (less than once a year), 2 = threat occurs 1 to 30 days/year, 3 = threat occurs 1 to 3 months/year, 4 = threat occurs 3 to 9 months/year, 5 = threat is near-continuous to continuous. HM = habitat modification, * = specific frequency based on climate change predictions (60 days/year).

| **Threats** | **Saltmarshes** | **Mangroves** | **Intertidal (soft)** | **Intertidal (rocky)** | **Seagrasses** | **Algal forest & rocky reef** | **Pelagic** | **Soft bottom** |
| --- | --- | --- | --- | --- | --- | --- | --- | --- |
| Acid sulphate soil disturbance | 1 | 1 | 1 | 1 | 1 | 0 | 0 | 0 |
| Aquaculture: mussels | 0 | 0 | 0 | 0 | 0 | 0 | 5 | 0 |
| Aquaculture: Pacific oyster | 0 | 0 | 5 | 0 | 5 | 0 | 0 | 0 |
| Aquaculture: predatory fish | 3 | 4 | 4 | 0 | 4 | 4 | 4 | 4 |
| Boating | 0 | 0 | 0 | 0 | 5 | 5 | 5 | 5 |
| Climate change: decrease in rainfall | 5 | 5 | 0 | 0 | 0 | 0 | 0 | 0 |
| Climate change: global warming | 5 | 5 | 5 | 5 | 5 | 5 | 5 | 5 |
| Climate change: increase in extreme rainfall | 1 | 1 | 1 | 1 | 1 | 1 | 1 | 1 |
| Climate change: increase in hot weather | * | * | * | * | * | * | * | * |
| Climate change: ocean acidification | 0 | 5 | 5 | 5 | 5 | 5 | 5 | 5 |
| Climate change: sea level rise | 5 | 5 | 5 | 5 | 5 | 5 | 0 | 0 |
| Coastal activities | 5 | 5 | 5 | 5 | 5 | 0 | 0 | 0 |
| Coastal habitat modification | 5 | 5 | 5 | 5 | 5 | 0 | 0 | 0 |
| Disease & pathogen outbreaks | 1 | 1 | 1 | 1 | 1 | 1 | 1 | 1 |
| Fishing: demersal trawl | 0 | 0 | 0 | 0 | 3 | 0 | 3 | 3 |
| Fishing: hand collection | 0 | 0 | 0 | 0 | 0 | 4 | 0 | 0 |
| Fishing: handline, longline | 0 | 0 | 4 | 0 | 4 | 4 | 4 | 4 |
| Fishing: haul nets, gillnets | 0 | 0 | 3 | 0 | 3 | 3 | 0 | 3 |
| Fishing: illegal | 0 | 2 | 3 | 2 | 2 | 2 | 2 | 2 |
| Fishing: pots | 0 | 0 | 3 | 0 | 3 | 4 | 0 | 3 |
| Fishing: purse seine | 0 | 0 | 0 | 0 | 0 | 0 | 4 | 0 |
| Harmful algal blooms | 0 | 1 | 1 | 1 | 1 | 1 | 1 | 1 |
| Invasive species: benthic filter-feeders | 0 | 5 | 5 | 5 | 5 | 5 | 0 | 5 |
| Invasive species: encrusting, fouling | 0 | 4 | 4 | 4 | 4 | 4 | 0 | 4 |
| Invasive species: predators, parasites | 0 | 5 | 5 | 5 | 5 | 5 | 0 | 5 |
| Marine HM: dredging | 1 | 1 | 1 | 1 | 1 | 1 | 1 | 1 |
| Marine HM: harbors, ports | 5 | 5 | 5 | 5 | 5 | 5 | 5 | 5 |
| Marine HM: jetties, seawalls | 5 | 5 | 5 | 5 | 5 | 5 | 5 | 5 |
| Marine HM: marinas, boat ramps | 5 | 5 | 5 | 5 | 5 | 0 | 5 | 5 |
| Pollution: brine discharge | 3 | 5 | 5 | 5 | 5 | 5 | 5 | 5 |
| Pollution: heavy metals | 5 | 5 | 5 | 5 | 5 | 5 | 5 | 5 |
| Pollution: marine debris | 5 | 5 | 5 | 5 | 5 | 5 | 5 | 5 |
| Pollution: nutrient discharge (point source) | 3 | 5 | 5 | 5 | 5 | 5 | 5 | 5 |
| Pollution: oil spill (100s of tonnes) | 1 | 1 | 1 | 1 | 1 | 1 | 1 | 1 |
| Pollution: sediment runoff & dust | 5 | 5 | 5 | 5 | 5 | 5 | 5 | 5 |
| Pollution: thermal | 3 | 5 | 5 | 0 | 5 | 0 | 0 | 0 |
| Shipping | 0 | 0 | 0 | 0 | 5 | 5 | 5 | 5 |
| Shipping (high level) | 0 | 0 | 0 | 0 | 5 | 5 | 5 | 5 |
