# Supplementary material for: Eight habitats, 38 threats and 55 experts: Assessing ecological risk in a multi-use marine region
Source: PLoS One. 2017 May 10;12(5):e0177393. doi: 10.1371/journal.pone.0177393 (PMC5425208; doi:10.1371/journal.pone.0177393)
Supplement: S3 Table — 0 = threat does not overlap with habitat, 1 = threat covers < 10% of habitat, 2 = threat covers 10 to 25% of habitat, 3 = threat covers 25 to 50% of habitat, 4 = threat covers 50 to 75% of habitat, 5 = threat covers 75 to 100% of habitat, dash = no available data. HM = habitat modification. Data quality category = adequate spatial data (1), limited spatial data or well-documented ‘whole-of-habitat’ threat (2), expert opinion/qualitative data (3), limited knowledge/no data (4), not applicable, near-future threat (NA). (DOCX) [file pone.0177393.s003.docx]

**Table S3.** Spatial exposure scores for each threat-habitat combination in Spencer Gulf. 0 = threat does not overlap with habitat, 1 = threat covers < 10% of habitat, 2 = threat covers 10 to 25% of habitat, 3 = threat covers 25 to 50% of habitat, 4 = threat covers 50 to 75% of habitat, 5 = threat covers 75 to 100% of habitat, dash = no available data. HM = habitat modification. Data quality category = adequate spatial data (1), limited spatial data or well-documented ‘whole-of-habitat’ threat (2), expert opinion/qualitative data (3), limited knowledge/no data (4), not applicable, near-future threat (NA).

| **Threats** | **Saltmarshes** | **Mangroves** | **Intertidal (soft)** | **Intertidal (rocky)** | **Seagrasses** | **Algal forest & rocky reef** | **Pelagic** | **Soft bottom** | **Data quality category** |
| --- | --- | --- | --- | --- | --- | --- | --- | --- | --- |
| Acid sulphate soil disturbance^a^ | 4 | 5 | 2 | 1 | 1 | 0 | 0 | 0 | 1 |
| Aquaculture: mussels | 0 | 0 | 0 | 0 | 0 | 0 | 1 | 0 | 1 |
| Aquaculture: Pacific oyster | 0 | 0 | 1 | 0 | 1 | 0 | 0 | 0 | 1 |
| Aquaculture: predatory fish | 1 | 1 | 1 | 0 | 2 | 1 | 1 | 1 | 2 |
| Boating | 0 | 0 | 0 | 0 | 4 | 4 | 4 | 5 | 2 |
| Climate change: decrease in rainfall | 5 | 5 | 0 | 0 | 0 | 0 | 0 | 0 | 2 |
| Climate change: global warming | 5 | 5 | 5 | 5 | 5 | 5 | 5 | 5 | 2 |
| Climate change: increase in extreme rainfall | 5 | 5 | 4 | 4 | 2 | 1 | 1 | 1 | 3 |
| Climate change: increase in hot weather | 5 | 5 | 5 | 5 | 3 | 3 | 2 | 1 | 2 |
| Climate change: ocean acidification | 0 | 5 | 5 | 5 | 5 | 5 | 5 | 5 | 2 |
| Climate change: sea level rise | - | - | - | - | - | - | - | - | 4 |
| Coastal activities | 3 | 4 | 4 | 5 | 1 | 0 | 0 | 0 | 1 |
| Coastal habitat modification | 2 | 1 | 1 | 3 | 1 | 0 | 0 | 0 | 1 |
| Disease & pathogen outbreaks | - | - | - | - | - | - | - | - | 4 |
| Fishing: demersal trawl | 0 | 0 | 0 | 0 | 2 | 0 | 3 | 4 | 1 |
| Fishing: hand collection | 0 | 0 | 0 | 0 | 0 | 4 | 0 | 0 | 1 |
| Fishing: handline, longline | 0 | 0 | 1 | 0 | 1 | 1 | 1 | 1 | 3 |
| Fishing: haul nets, gillnets | 0 | 0 | 1 | 0 | 1 | 1 | 0 | 1 | 3 |
| Fishing: illegal | - | - | - | - | - | - | - | - | 4 |
| Fishing: pots | 0 | 0 | 1 | 0 | 1 | 4 | 0 | 1 | 1 and 3 |
| Fishing: purse seine | 0 | 0 | 0 | 0 | 0 | 0 | 4 | 0 | 1 |
| Harmful algal blooms | - | - | - | - | - | - | - | - | 4 |
| Invasive species: benthic filter-feeders | 0 | 1 | 1 | 1 | 1 | 1 | 0 | 1 | 2 |
| Invasive species: encrusting, fouling | 0 | 1 | 1 | 1 | 1 | 1 | 0 | 1 | 2 |
| Invasive species: predators, parasites | 0 | 1 | 1 | 1 | 1 | 1 | 0 | 1 | 2 |
| Marine HM: dredging |  |  |  |  |  |  |  |  | N/A |
| Marine HM: harbors, ports | 1 | 2 | 1 | 1 | 1 | 1 | 1 | 1 | 1 |
| Marine HM: jetties, seawalls | 2 | 2 | 1 | 1 | 1 | 1 | 1 | 1 | 1 |
| Marine HM: marinas, boat ramps | 1 | 1 | 1 | 1 | 1 | 0 | 1 | 1 | 1 |
| Pollution: brine discharge ^ |  |  |  |  |  |  |  |  | N/A |
| Pollution: heavy metals | 4 | 4 | 3 | 2 | 2 | 1 | 1 | 1 | 2 |
| Pollution: marine debris | - | - | - | - | - | - | - | - | 4 |
| Pollution: nutrient discharge (point source) | 2 | 4 | 3 | 3 | 3 | 2 | 1 | 1 | 2 |
| Pollution: oil spill (100s of tonnes) ^b^ | 1 | 2 | 2 | 1 | 2 | 1 | 1 | 1 | 2 |
| Pollution: sediment runoff & dust | - | - | - | - | - | - | - | - | 4 |
| Pollution: thermal | 1 | 1 | 1 | 0 | 1 | 0 | 0 | 0 | 1 |
| Shipping | 0 | 0 | 0 | 0 | 2 | 2 | 3 | 3 | 1 |
| Shipping (high level) ^ | - | - | - | - | - | - | - | - | N/A |

^a^ level of spatial exposure includes both undisturbed and disturbed soil, ^b^ spatial exposure is defined in this case as where an oil spill is most likely to occur and not the exposure of a single spill
