# Supplementary material for: Eight habitats, 38 threats and 55 experts: Assessing ecological risk in a multi-use marine region
Source: PLoS One. 2017 May 10;12(5):e0177393. doi: 10.1371/journal.pone.0177393 (PMC5425208; doi:10.1371/journal.pone.0177393)
Supplement: S4 Table — Statistics below are based on number of surveys and percentage of the total number (n = 81). (DOCX) [file pone.0177393.s004.docx]

**Table S4.** Background information associated with survey respondents (n = 55). Statistics below are based on number of surveys and percentage of the total number (n = 81).

| **Background attribute** | **number** | **%** |
| --- | --- | --- |
|  |  |  |
| **Level of experience (Spencer Gulf)** |  |  |
| Little or no knowledge of SA’s gulf environments | 0 | 0 |
| A general understanding of SA’s gulf environments | 18 | 22 |
| Some work experience in Spencer Gulf | 51 | 63 |
| Extensive work experience in Spencer Gulf | 12 | 15 |
|  |  |  |
| **Bioregion focus within Spencer Gulf** |  |  |
| None specified | 60 | 74 |
| North | 5 | 6 |
| Central | 0 | 0 |
| South | 9 | 11 |
| North and central | 0 | 0 |
| South and central | 3 | 4 |
| North and south | 4 | 5 |
|  |  |  |
| **Level of experience (habitat)** |  |  |
| < 2 years | 4 | 5 |
| 2 - 5 years | 20 | 25 |
| 5 - 10 years | 19 | 23 |
| 10 - 20 years | 22 | 27 |
| > 20 years | 16 | 20 |
|  |  |  |
| **Type of position** |  |  |
| Academic (postgraduate) | 7 | 9 |
| Academic (staff) | 26 | 32 |
| Consultant | 5 | 6 |
| State government | 35 | 43 |
| Other | 8 | 10 |
|  |  |  |
| **Primary work responsibility** |  |  |
| Consultancy | 3 | 4 |
| Environmental policy | 1 | 1 |
| Natural resource or environmental management | 10 | 12 |
| Research | 61 | 75 |
| Other | 6 | 7 |
