# Supplementary material for: Eight habitats, 38 threats and 55 experts: Assessing ecological risk in a multi-use marine region
Source: PLoS One. 2017 May 10;12(5):e0177393. doi: 10.1371/journal.pone.0177393 (PMC5425208; doi:10.1371/journal.pone.0177393)
Supplement: S1 Fig — Each point represents the mean or standard error of the effect score for each threat-habitat combination and the grey horizontal line represents the grand mean of those points. (DOCX) [file pone.0177393.s005.docx]

**a**

**b**

**Figure S1.** Level of variance in survey response values (raw effect scores, most-likely scenario) among groups (a; mean) and within groups (b; standard error) for each habitat, with habitats ordered from highest sample size (seagrass; n = 15) to lowest (mangroves; n = 6). Each point represents the mean or standard error of the effect score for each threat-habitat combination and the grey horizontal line represents the grand mean of those points.
