# Supplementary material for: Eight habitats, 38 threats and 55 experts: Assessing ecological risk in a multi-use marine region
Source: PLoS One. 2017 May 10;12(5):e0177393. doi: 10.1371/journal.pone.0177393 (PMC5425208; doi:10.1371/journal.pone.0177393)
Supplement: S2 Fig — Left-hand graph: effect scores for each threat and scenario. Scenario effect scores are indicated by vertical black dashes, ranging from best-case (yellow), most-likely and worst-case (red). Uncertainty scores (effect scoreworst minus effect scorebest) for each threat are indicated by crosses. Threats are ranked from the highest to lowest most-likely effect. Near-future threats (^) and threats lacking spatial information (*) are not included in the final risk analysis. Right-hand graph: risk scores for each threat based on most-likely scenario, ranked from highest to lowest risk. Numbers relate to data quality category of the spatial information: adequate spatial data (1), limited spatial data or well-documented ‘whole-of-habitat’ threat (2), expert opinion/qualitative data (3), limited knowledge/no data (4). (DOCX) [file pone.0177393.s006.docx]

**Figure S2.** Risk assessment result for each habitat. **Left-hand graph:** effect scores for each threat and scenario. Scenario effect scores are indicated by vertical black dashes, ranging from best-case (yellow), most-likely and worst-case (red). Uncertainty scores (effect score_worst_ minus effect score_best_) for each threat are indicated by crosses. Threats are ranked from the highest to lowest most-likely effect. Near-future threats (^) and threats lacking spatial information (*) are not included in the final risk analysis. **Right-hand graph:** risk scores for each threat based on most-likely scenario, ranked from highest to lowest risk. Numbers relate to data quality category of the spatial information: adequate spatial data (1), limited spatial data or well-documented ‘whole-of-habitat’ threat (2), expert opinion/qualitative data (3), limited knowledge/no data (4).

**Seagrasses**

**Soft bottom habitats**

**Pelagic habitats**

**Algal forest & rocky reef**

**Saltmarshes**

**Intertidal (soft unvegetated substrate)**

**Intertidal (rocky substrate)**

**Mangroves**
