# Supplementary material for: Eight habitats, 38 threats and 55 experts: Assessing ecological risk in a multi-use marine region
Source: PLoS One. 2017 May 10;12(5):e0177393. doi: 10.1371/journal.pone.0177393 (PMC5425208; doi:10.1371/journal.pone.0177393)
Supplement: S1 File — Includes Participant Information Sheet and Survey Reference Sheet. (DOCX) [file pone.0177393.s007.docx]

**PARTICIPANT INFORMATION SHEET**

**Spencer Gulf Ecosystem Development Initiative**

Assessing ecological risk of multiple threats to marine habitats in Spencer Gulf

**Summary**

We would like to invite you to participate in a survey conducted by researchers at The University of Adelaide. Using expert opinion, the aim of the survey is to determine the level of ecological risk multiple threats pose to different marine habitats in Spencer Gulf (South Australia). The methodology has undergone extensive review and consultation by relevant experts from both academia and government. Please read the information below before commencing the survey. Your input and time is much appreciated and essential to the success of the project.

**Project overview**

South Australia, and in particular the Spencer Gulf region, has significant opportunities for expansion of mining, with a large number of new mineral extraction and processing ventures proposed. Associated with this expansion will be increased shipping, port development and potentially biosecurity risks. Spencer Gulf is also recognised for its clean, green image and high quality seafood production, as well as its numerous environmental assets, iconic and endangered species, and ecotourism ventures. The key issue, therefore, is how South Australia can balance the development of mining ventures, expansion of fishing and aquaculture, and conservation and recreation needs, while simultaneously delivering on the environmental, social and economic objectives associated with the management and utilisation of Spencer Gulf. To address this issue an **integrated approach to marine management** is required to ensure that the ecological, economic and social outcomes are optimised across industries and user groups for the benefit of all South Australians, while preserving the integrity of the ecosystem.

The **Spencer Gulf Ecosystem Development Initiative** (SGEDI), led by Prof Gillanders, is a major, multi-year initiative designed to develop knowledge and tools to inform integrated management of Spencer Gulf (see <http://www.gillanderslab.org/#!spencer-gulf/c19xd> for more details). This ecological risk assessment is an integral component of SGEDI and data generated from this project will also be used in a subsequent study to generate spatially-resolved maps of *cumulative* impacts to Spencer Gulf.

**Project aims**

Our aim is to determine the level of risk multiple human-mediated threats pose to different marine habitats in Spencer Gulf. This will be achieved using a comprehensive, repeatable and transparent framework consisting largely of the attached survey. Using your survey data, we aim to:

1. Quantitatively rank the relative risk of marine habitats to multiple threats, as well as identify level of uncertainty associated with each ranking
2. Identify habitats most at risk to current threats and threats that pose the greatest risk to Spencer Gulf
3. Identify key knowledge gaps associated with each habitats and each threat
4. Provide stakeholders with access to independent and credible information about Spencer Gulf
5. Inform future research and management priorities in Spencer Gulf

**Who is undertaking the project?**

The project is led by Prof Bronwyn Gillanders (University of Adelaide). Other members of the research team include Dr Zoë Doubleday, Dr Alice Jones (University of Adelaide) and A/Prof Tim Ward (South Australian Research and Development Institute, Aquatic Sciences). SGEDI is funded via a consortium of industry including BHP Billiton, Santos, Arrium, Centrex, Flinders Ports, Alinta and Nyrstar.

**Why am I being invited to participate?**

You have been identified as an expert for one or more marine habitats in Spencer Gulf. As such, we believe you would be a suitable candidate to assess the relative vulnerability of your habitat to a range of threats in the region. However, if we have misinterpreted your expertise, the survey, is of course, voluntary and you can skip/leave blank any questions you feel you cannot answer.

**What will I be asked to do?**

You will be asked to undertake a survey, the bulk of which involves assessing the relative risk of your chosen habitat to multiple anthropogenic threats (Part III). The assessment is highly-structured and quantitative (i.e. score-based), however, there will be opportunity for you to provide comments if desired.

The survey includes the following components

Part I: Background and work experience

Part II: Listing top five threats to your habitat

Part III: Assessing the ecological consequence of each threat to your habitat

Part IV: Assessing natural recovery potential of your habitat to threat disturbance

Part V: Assessing synergistic interactions among threats

Part VI: Feedback

There is a different survey for each habitat. Each survey is the same apart from the combination of threats that you will assess. You can assess as many habitats as you like that are relevant to your expertise. One survey will take **about 30 minutes** to complete (less for subsequent surveys). If you do not complete the survey in one session, it can be saved and completed at another time.

Instructions are provided throughout the survey, along with an attached **survey reference sheet**, which we recommend you print out or have open on another screen before commencing. However, if any clarifications are needed please contact [Zoe Doubleday](mailto:zoe.doubleday@adelaide.edu.au).

You can choose to do the survey **online**, on a **printed hardcopy** or via a **face-to-face interview**.

**Can I withdraw from the project?**

Participation in this project is completely voluntary. Please note that **by completing the survey you give your consent to take part in this project.** However, you can withdraw from the project at any time. If you wish to withdraw your survey response after completion of the survey, you will be able to do so prior to the data being compiled and analysed. Please contact us if you have any concerns regarding this.

**What will happen to my information?**

Your individual survey response and corresponding identity will be confidential. Each participant will be given a random identifier code so that analyses are conducted without personal identifying information attached. Results (based on aggregated data and/or raw anonymous data) will be published in scientific journals and reports and presented at a range of forums (e.g. scientific conferences, stakeholder meetings, media). Survey data may also be used in future studies and handled in a similar manner as above. You will be asked in the survey whether or not you wish to be identified as a contributing expert in resultant publications and also whether you wish to be kept informed of resultant publications. Data collected from the survey will be securely stored on the University of Adelaide server.

**Ethical considerations and project risks**

The study has been approved by the Human Research Ethics Committee at the University of Adelaide (approval number **H-2015-234**). Apart from the time it will take to complete the survey, there are no foreseeable risks or burdens associated with the project. The survey has been kept as succinct as possible (while still maintaining project integrity) and has been tested for readability by the research team.

However, if you have questions or problems associated with your participation in the project, or wish to raise a concern or complaint about the project, then you should consult the [Principal Investigator](mailto:bronwyn.gillanders@adelaide.edu.au). If you wish to speak with an independent person regarding concerns or a complaint, the University’s policy on research involving human participants, or your rights as a participant please contact the Human Research Ethics Committee’s Secretariat on phone +61 8 8313 6028 or by email to [hrec@adelaide.edu.au](mailto:hrec@adelaide.edu.au). Any complaint or concern will be treated in confidence and fully investigated. You will be informed of the outcome.

**Contact details**

**Primary Contacts**

Prof Bronwyn Gillanders (Principal Investigator) – please contact for general inquiries

University of Adelaide

Ph: 08 83136235; Email: [bronwyn.gillanders@adelaide.edu.au](mailto:bronwyn.gillanders@adelaide.edu.au)

Dr Zoë Doubleday – please contact for specific queries regarding the survey

University of Adelaide

Ph: 08 83136587; Email: [zoe.doubleday@adelaide.edu.au](mailto:zoe.doubleday@adelaide.edu.au)

**Other Contacts**

A/Prof Tim Ward

South Australian Research & Development Institute (SARDI) - Aquatic Sciences

PH: 08 82075433; Email: [Tim.Ward@sa.gov.au](mailto:Tim.Ward@sa.gov.au)

Dr Alice Jones

University of Adelaide

Ph: 08 83132243; Email: [alice.jones01@adelaide.edu.au](mailto:alice.jones01@adelaide.edu.au)

# Spencer Gulf Risk Assessment:

# [specified habitat]

###

###

### For project background, human ethics, and contact details please download Participant Information Sheet

### This study has been approved by the Human Research Ethics Committee at the University of Adelaide (H-2015-234)

**Key Definitions**

### Region: Spencer Gulf (30,000 km2)
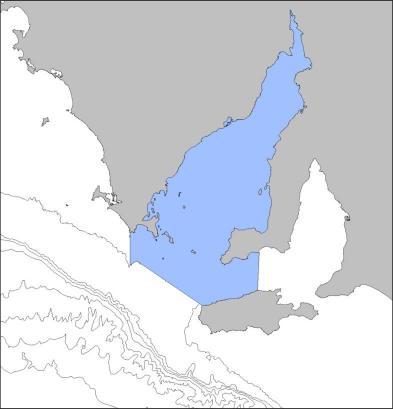
 Habitat: physical attributes and all biological constituents (e.g. populations, species) Threats: the survey is focused on present-day threats, and, where indicated, near-future threats (e.g. climate change threats are based on near-future projections)

**Part I: Background and Experience**

### Name

_________________________________________________

### Email address (required)*

_________________________________________________

### Affiliation

_________________________________________________

#### Type of position

( ) Academic (staff)

( ) Academic (postgraduate student)

( ) Consultant

( ) Government agency (federal)

( ) Government agency (state)

( ) Non-governmental organisation

( ) Other: _________________________________________________

#### Primary work responsibility

( ) Consultancy

( ) Environmental Policy

( ) Natural Resource Management

( ) Research

( ) Other: _________________________________________________

#### Years of experience with relevant habitat:

( ) less than 2 years

( ) 2 to 5 years

( ) 5 to 10 years

( ) 10 to 20 years

( ) more than 20 years

#### Please indicate what best describes your experience/knowledge of Spencer Gulf

( ) I have extensive work/research experience in Spencer Gulf

( ) I have some work/research experience in Spencer Gulf

( ) I have a general understanding of South Australia's gulf environments

( ) I have little or no knowledge of South Australia's gulf environments

( ) Other: _________________________________________________

#### ****Only if relevant,**** please indicate if your expertise is especially focused on 1 or 2 bioregions in Spencer Gulf.

[ ] North

[ ] Central

[ ] South

### Spencer Gulf bioregions (total area: 30,000 km2)

**Part II: Listing top five threats to your habitat**

#### This is NOT the main part of the survey, please only take a few minutes to complete.

#### Rank the top 5 threats that you think are currently impacting your habitat the most (1 = greatest impact)

#### For more details on each threat, please download ****detailed Threat List****

________Acid sulphate soil disturbance

________Aquaculture: mussels

________Aquaculture: Pacific oyster

________Aquaculture: predatory fish (+ diffuse nutrients)

________Boating

________Brine discharge

________Climate change: ocean acidification

________Climate change: global warming

________Climate change: sea level rise

________Climate change: increase in hot weather events

________Climate change: increase in extreme rainfall events

________Climate change: decrease in rainfall

________Coastal activities

________Coastal habitat modification

________Disease & pathogens

________Fishing: hand collection

________Fishing: handline, longline

________Fishing: haul nets, gillnets

________Fishing: pots

________Fishing: demersal trawl

________Fishing: purse seine

________Fishing: illegal

________Harmful algal blooms

________Heavy metals

________Invasive sp: benthic filter-feeders

________Invasive sp: encrusting, fouling

________Invasive sp: predators, parasites

________Marine debris

________Marine habitat modification: commercial harbors, ports

________Marine habitat modification: jetties, seawalls

________Marine habitat modification: marinas, boat ramps

________Marine habitat modification: dredging

________Nutrient discharge (point source)

________Oil spill, size: 100s of tonnes

________Sediment runoff & dust

________Shipping

________Thermal pollution

### Are there any threats that you would list in the top five that are not listed here? If yes, how would they rank among the top five threats?

____________________________________________

____________________________________________

____________________________________________

____________________________________________

**Part III: Assessing the ecological consequence of each threat to your habitat**

- This IS the main part of the survey and will take the longest to complete.
- Please download **Survey Reference Sheet** [attached here at end of survey] before commencing. It is recommended that you print this out or keep open on another screen for quick reference while undertaking the survey.
- There are ## threats to assess for [specified habitat]. The order of threats is randomised per participant. *Note this is an example survey listing all threats that were assessed for all habitats, with the number of threats varying from habitat to habitat.*

#### ****Acid sulphate soil disturbance****

#### Also includes acid water drainage from ASS disturbance

The average frequency this threat occurs at this habitat

- rare (less than once a year)

Consequence of this threat

- See **Survey Reference Sheet** for detailed instructions and definitions
- Select 1 number per row. 0 = no/minimal consequence, 4 = high consequence
- The scores for each scenario can be very different (e.g. 0, 2 and 4) if you are highly uncertain of the consequence or the same (e.g. 2, 2 and 2) if you are highly certain of the consequence

|  | **0** | **1** | **2** | **3** | **4** |
| --- | --- | --- | --- | --- | --- |
| Change in physical habitat structure (best-case scenario) | ( ) | ( ) | ( ) | ( ) | ( ) |
| Change in physical habitat structure (most-likely scenario) | ( ) | ( ) | ( ) | ( ) | ( ) |
| Change in physical habitat structure (worst-case scenario) | ( ) | ( ) | ( ) | ( ) | ( ) |
| Change in species composition and trophic structure (best-case scenario) | ( ) | ( ) | ( ) | ( ) | ( ) |
| Change in species composition and trophic structure (most-likely scenario) | ( ) | ( ) | ( ) | ( ) | ( ) |
| Change in species composition and trophic structure (worst-case scenario) | ( ) | ( ) | ( ) | ( ) | ( ) |

#### Comments:

#### Aquaculture: mussels

#### growout, sub-tidal sub-surface longlines

The average frequency this threat occurs at this habitat

- continuous

Consequence of this threat

- See **Survey Reference Sheet** for detailed instructions and definitions
- Select 1 number per row. 0 = no/minimal consequence, 4 = high consequence
- The scores for each scenario can be very different (e.g. 0, 2 and 4) if you are highly uncertain of the consequence or the same (e.g. 2, 2 and 2) if you are highly certain of the consequence

|  | **0** | **1** | **2** | **3** | **4** |
| --- | --- | --- | --- | --- | --- |
| Change in physical habitat structure (best-case scenario) | ( ) | ( ) | ( ) | ( ) | ( ) |
| Change in physical habitat structure (most-likely scenario) | ( ) | ( ) | ( ) | ( ) | ( ) |
| Change in physical habitat structure (worst-case scenario) | ( ) | ( ) | ( ) | ( ) | ( ) |
| Change in species composition and trophic structure (best-case scenario) | ( ) | ( ) | ( ) | ( ) | ( ) |
| Change in species composition and trophic structure (most-likely scenario) | ( ) | ( ) | ( ) | ( ) | ( ) |
| Change in species composition and trophic structure (worst-case scenario) | ( ) | ( ) | ( ) | ( ) | ( ) |

#### Comments:

#### ****Aquaculture: Pacific oyster****

#### growout, intertidal rack-and-rail and longlines

#### sub-threats include trampling, sedimentation, shading with rack-and-rail

#### does not include impacts from invasive feral populations or hatcheries

The average frequency this threat occurs at this habitat

- continuous

Consequence of this threat

- See **Survey Reference Sheet** for detailed instructions and definitions
- Select 1 number per row. 0 = no/minimal consequence, 4 = high consequence
- The scores for each scenario can be very different (e.g. 0, 2 and 4) if you are highly uncertain of the consequence or the same (e.g. 2, 2 and 2) if you are highly certain of the consequence

|  | **0** | **1** | **2** | **3** | **4** |
| --- | --- | --- | --- | --- | --- |
| Change in physical habitat structure (best-case scenario) | ( ) | ( ) | ( ) | ( ) | ( ) |
| Change in physical habitat structure (most-likely scenario) | ( ) | ( ) | ( ) | ( ) | ( ) |
| Change in physical habitat structure (worst-case scenario) | ( ) | ( ) | ( ) | ( ) | ( ) |
| Change in species composition and trophic structure (best-case scenario) | ( ) | ( ) | ( ) | ( ) | ( ) |
| Change in species composition and trophic structure (most-likely scenario) | ( ) | ( ) | ( ) | ( ) | ( ) |
| Change in species composition and trophic structure (worst-case scenario) | ( ) | ( ) | ( ) | ( ) | ( ) |

#### Comments:

#### ****Aquaculture: predatory fish (includes diffuse nutrients)****

#### growout, sea cages, southern bluefin tuna and yellowtail kingfish

#### main threat includes high diffuse nutrient input

#### does not include impacts from hatcheries or introduced pathogens

The average frequency this threat occurs at this habitat

- 3 to 9 months/year

Consequence of this threat

- See **Survey Reference Sheet** for detailed instructions and definitions
- Select 1 number per row. 0 = no/minimal consequence, 4 = high consequence
- The scores for each scenario can be very different (e.g. 0, 2 and 4) if you are highly uncertain of the consequence or the same (e.g. 2, 2 and 2) if you are highly certain of the consequence

|  | **0** | **1** | **2** | **3** | **4** |
| --- | --- | --- | --- | --- | --- |
| Change in physical habitat structure (best-case scenario) | ( ) | ( ) | ( ) | ( ) | ( ) |
| Change in physical habitat structure (most-likely scenario) | ( ) | ( ) | ( ) | ( ) | ( ) |
| Change in physical habitat structure (worst-case scenario) | ( ) | ( ) | ( ) | ( ) | ( ) |
| Change in species composition and trophic structure (best-case scenario) | ( ) | ( ) | ( ) | ( ) | ( ) |
| Change in species composition and trophic structure (most-likely scenario) | ( ) | ( ) | ( ) | ( ) | ( ) |
| Change in species composition and trophic structure (worst-case scenario) | ( ) | ( ) | ( ) | ( ) | ( ) |

#### Comments:

#### Brine discharge

#### from a desalination plant

#### while no desalination plants currently exist in Spencer Gulf, it is expected that they will be developed in the near-future

The average frequency this threat occurs at this habitat

- continuous

Consequence of this threat

- See **Survey Reference Sheet** for detailed instructions and definitions
- Select 1 number per row. 0 = no/minimal consequence, 4 = high consequence
- The scores for each scenario can be very different (e.g. 0, 2 and 4) if you are highly uncertain of the consequence or the same (e.g. 2, 2 and 2) if you are highly certain of the consequence

|  | **0** | **1** | **2** | **3** | **4** |
| --- | --- | --- | --- | --- | --- |
| Change in physical habitat structure (best-case scenario) | ( ) | ( ) | ( ) | ( ) | ( ) |
| Change in physical habitat structure (most-likely scenario) | ( ) | ( ) | ( ) | ( ) | ( ) |
| Change in physical habitat structure (worst-case scenario) | ( ) | ( ) | ( ) | ( ) | ( ) |
| Change in species composition and trophic structure (best-case scenario) | ( ) | ( ) | ( ) | ( ) | ( ) |
| Change in species composition and trophic structure (most-likely scenario) | ( ) | ( ) | ( ) | ( ) | ( ) |
| Change in species composition and trophic structure (worst-case scenario) | ( ) | ( ) | ( ) | ( ) | ( ) |

#### Comments:

#### ****Boating****

#### from recreational activities, commercial fishing, aquaculture operations and ecotourism

#### sub-threats include grounding, sediment re-suspension, anchor damage, waste discharge and antifoulants

#### does not include threats related to invasive species or fishing

The average frequency this threat occurs at this habitat

- continuous

Consequence of this threat

- See **Survey Reference Sheet** for detailed instructions and definitions
- Select 1 number per row. 0 = no/minimal consequence, 4 = high consequence
- The scores for each scenario can be very different (e.g. 0, 2 and 4) if you are highly uncertain of the consequence or the same (e.g. 2, 2 and 2) if you are highly certain of the consequence

|  | **0** | **1** | **2** | **3** | **4** |
| --- | --- | --- | --- | --- | --- |
| Change in physical habitat structure (best-case scenario) | ( ) | ( ) | ( ) | ( ) | ( ) |
| Change in physical habitat structure (most-likely scenario) | ( ) | ( ) | ( ) | ( ) | ( ) |
| Change in physical habitat structure (worst-case scenario) | ( ) | ( ) | ( ) | ( ) | ( ) |
| Change in species composition and trophic structure (best-case scenario) | ( ) | ( ) | ( ) | ( ) | ( ) |
| Change in species composition and trophic structure (most-likely scenario) | ( ) | ( ) | ( ) | ( ) | ( ) |
| Change in species composition and trophic structure (worst-case scenario) | ( ) | ( ) | ( ) | ( ) | ( ) |

#### Comments:

#### ****Climate change: ocean acidification (2015 - 2030)****

#### decrease in ocean pH by 0.07

#### consider that ocean pH has already decreased over the past 200 years by about 0.1

The average frequency this threat occurs at this habitat

- continuous

Consequence of this threat

- See **Survey Reference Sheet** for detailed instructions and definitions
- Select 1 number per row. 0 = no/minimal consequence, 4 = high consequence
- The scores for each scenario can be very different (e.g. 0, 2 and 4) if you are highly uncertain of the consequence or the same (e.g. 2, 2 and 2) if you are highly certain of the consequence

|  | **0** | **1** | **2** | **3** | **4** |
| --- | --- | --- | --- | --- | --- |
| Change in physical habitat structure (best-case scenario) | ( ) | ( ) | ( ) | ( ) | ( ) |
| Change in physical habitat structure (most-likely scenario) | ( ) | ( ) | ( ) | ( ) | ( ) |
| Change in physical habitat structure (worst-case scenario) | ( ) | ( ) | ( ) | ( ) | ( ) |
| Change in species composition and trophic structure (best-case scenario) | ( ) | ( ) | ( ) | ( ) | ( ) |
| Change in species composition and trophic structure (most-likely scenario) | ( ) | ( ) | ( ) | ( ) | ( ) |
| Change in species composition and trophic structure (worst-case scenario) | ( ) | ( ) | ( ) | ( ) | ( ) |

#### Comments:

#### ****Climate change: global warming (2015 - 2030)****

#### ****For inter-tidal and sub-tidal habitats:****

#### increase in mean sea surface temperature by 0.8°C

#### consider that SST increase, over the past 100 years, has been relatively minimal for the South Australian region (< 0.1°C)

#### ****For inter-tidal and extra-tidal habitats:****

#### increase in mean air temperature by 0.4 to 1.1°C

#### consider that air temperature, over the past 100 years, has already increased by around 0.7°C in the South Australian region

The average frequency this threat occurs at this habitat

- continuous

Consequence of this threat

- See **Survey Reference Sheet** for detailed instructions and definitions
- Select 1 number per row. 0 = no/minimal consequence, 4 = high consequence
- The scores for each scenario can be very different (e.g. 0, 2 and 4) if you are highly uncertain of the consequence or the same (e.g. 2, 2 and 2) if you are highly certain of the consequence

|  | **0** | **1** | **2** | **3** | **4** |
| --- | --- | --- | --- | --- | --- |
| Change in physical habitat structure (best-case scenario) | ( ) | ( ) | ( ) | ( ) | ( ) |
| Change in physical habitat structure (most-likely scenario) | ( ) | ( ) | ( ) | ( ) | ( ) |
| Change in physical habitat structure (worst-case scenario) | ( ) | ( ) | ( ) | ( ) | ( ) |
| Change in species composition and trophic structure (best-case scenario) | ( ) | ( ) | ( ) | ( ) | ( ) |
| Change in species composition and trophic structure (most-likely scenario) | ( ) | ( ) | ( ) | ( ) | ( ) |
| Change in species composition and trophic structure (worst-case scenario) | ( ) | ( ) | ( ) | ( ) | ( ) |

#### Comments:

#### ****Climate change: increase in extreme rainfall events (2015 to 2030)****

#### Increase in the intensity, rather than frequency, of rainfall events. Magnitude of increase unknown.

The average frequency this threat occurs at this habitat

- 1 to 30 days/year

Consequence of this threat

- See **Survey Reference Sheet** for detailed instructions and definitions
- Select 1 number per row. 0 = no/minimal consequence, 4 = high consequence
- The scores for each scenario can be very different (e.g. 0, 2 and 4) if you are highly uncertain of the consequence or the same (e.g. 2, 2 and 2) if you are highly certain of the consequence

|  | **0** | **1** | **2** | **3** | **4** |
| --- | --- | --- | --- | --- | --- |
| Change in physical habitat structure (best-case scenario) | ( ) | ( ) | ( ) | ( ) | ( ) |
| Change in physical habitat structure (most-likely scenario) | ( ) | ( ) | ( ) | ( ) | ( ) |
| Change in physical habitat structure (worst-case scenario) | ( ) | ( ) | ( ) | ( ) | ( ) |
| Change in species composition and trophic structure (best-case scenario) | ( ) | ( ) | ( ) | ( ) | ( ) |
| Change in species composition and trophic structure (most-likely scenario) | ( ) | ( ) | ( ) | ( ) | ( ) |
| Change in species composition and trophic structure (worst-case scenario) | ( ) | ( ) | ( ) | ( ) | ( ) |

#### Comments:

**Climate change: decrease in rainfall (2015 to 2030)**

- **15% decrease in winter rainfall**
- **consider that there has been a prolonged period of drying in the South Australian region since the 1990s**

The average frequency this threat occurs at this habitat

- continuous

Consequence of this threat

- See **Survey Reference Sheet** for detailed instructions and definitions
- Select 1 number per row. 0 = no/minimal consequence, 4 = high consequence
- The scores for each scenario can be very different (e.g. 0, 2 and 4) if you are highly uncertain of the consequence or the same (e.g. 2, 2 and 2) if you are highly certain of the consequence

|  | **0** | **1** | **2** | **3** | **4** |
| --- | --- | --- | --- | --- | --- |
| Change in physical habitat structure (best-case scenario) | ( ) | ( ) | ( ) | ( ) | ( ) |
| Change in physical habitat structure (most-likely scenario) | ( ) | ( ) | ( ) | ( ) | ( ) |
| Change in physical habitat structure (worst-case scenario) | ( ) | ( ) | ( ) | ( ) | ( ) |
| Change in species composition and trophic structure (best-case scenario) | ( ) | ( ) | ( ) | ( ) | ( ) |
| Change in species composition and trophic structure (most-likely scenario) | ( ) | ( ) | ( ) | ( ) | ( ) |
| Change in species composition and trophic structure (worst-case scenario) | ( ) | ( ) | ( ) | ( ) | ( ) |

#### Comments:

#### ****Climate change: increase in hot weather events (2015 to 2030)****

#### increase in number of hot days (air temperature > 35°C) to 60 days/year

#### consider that most of the Spencer Gulf region has experienced about 30 hot days/year over the past 30 years

The average frequency this threat occurs at this habitat

- as detailed above

Consequence of this threat

- See **Survey Reference Sheet** for detailed instructions and definitions
- Select 1 number per row. 0 = no/minimal consequence, 4 = high consequence
- The scores for each scenario can be very different (e.g. 0, 2 and 4) if you are highly uncertain of the consequence or the same (e.g. 2, 2 and 2) if you are highly certain of the consequence

|  | **0** | **1** | **2** | **3** | **4** |
| --- | --- | --- | --- | --- | --- |
| Change in physical habitat structure (best-case scenario) | ( ) | ( ) | ( ) | ( ) | ( ) |
| Change in physical habitat structure (most-likely scenario) | ( ) | ( ) | ( ) | ( ) | ( ) |
| Change in physical habitat structure (worst-case scenario) | ( ) | ( ) | ( ) | ( ) | ( ) |
| Change in species composition and trophic structure (best-case scenario) | ( ) | ( ) | ( ) | ( ) | ( ) |
| Change in species composition and trophic structure (most-likely scenario) | ( ) | ( ) | ( ) | ( ) | ( ) |
| Change in species composition and trophic structure (worst-case scenario) | ( ) | ( ) | ( ) | ( ) | ( ) |

#### Comments:

#### ****Climate change: sea level rise (2015 to 2030)****

#### rise in mean sea level by 120 mm (12 cm)

#### consider that sea level has been rising along the Australian coastline by about 2.1 mm/year over the past 50 years

#### sub-threat includes ****increases in extreme sea level events**** (e.g. astronomical tides, storm surges and wind waves)

The average frequency this threat occurs at this habitat

- continuous

Consequence of this threat

- See **Survey Reference Sheet** for detailed instructions and definitions
- Select 1 number per row. 0 = no/minimal consequence, 4 = high consequence
- The scores for each scenario can be very different (e.g. 0, 2 and 4) if you are highly uncertain of the consequence or the same (e.g. 2, 2 and 2) if you are highly certain of the consequence

|  | **0** | **1** | **2** | **3** | **4** |
| --- | --- | --- | --- | --- | --- |
| Change in physical habitat structure (best-case scenario) | ( ) | ( ) | ( ) | ( ) | ( ) |
| Change in physical habitat structure (most-likely scenario) | ( ) | ( ) | ( ) | ( ) | ( ) |
| Change in physical habitat structure (worst-case scenario) | ( ) | ( ) | ( ) | ( ) | ( ) |
| Change in species composition and trophic structure (best-case scenario) | ( ) | ( ) | ( ) | ( ) | ( ) |
| Change in species composition and trophic structure (most-likely scenario) | ( ) | ( ) | ( ) | ( ) | ( ) |
| Change in species composition and trophic structure (worst-case scenario) | ( ) | ( ) | ( ) | ( ) | ( ) |

#### Comments:

#### ****Coastal activities****

#### such as walking, sunbathing, off-road vehicles and grazing

The average frequency this threat occurs at this habitat

- continuous

Consequence of this threat

- See **Survey Reference Sheet** for detailed instructions and definitions
- Select 1 number per row. 0 = no/minimal consequence, 4 = high consequence
- The scores for each scenario can be very different (e.g. 0, 2 and 4) if you are highly uncertain of the consequence or the same (e.g. 2, 2 and 2) if you are highly certain of the consequence

|  | **0** | **1** | **2** | **3** | **4** |
| --- | --- | --- | --- | --- | --- |
| Change in physical habitat structure (best-case scenario) | ( ) | ( ) | ( ) | ( ) | ( ) |
| Change in physical habitat structure (most-likely scenario) | ( ) | ( ) | ( ) | ( ) | ( ) |
| Change in physical habitat structure (worst-case scenario) | ( ) | ( ) | ( ) | ( ) | ( ) |
| Change in species composition and trophic structure (best-case scenario) | ( ) | ( ) | ( ) | ( ) | ( ) |
| Change in species composition and trophic structure (most-likely scenario) | ( ) | ( ) | ( ) | ( ) | ( ) |
| Change in species composition and trophic structure (worst-case scenario) | ( ) | ( ) | ( ) | ( ) | ( ) |

#### Comments:

#### ****Coastal habitat modification****

#### such as land reclamation, native vegetation removal and shoreline hardening

#### does not include threats associated with increased coastal activities, sediment runoff and acid sulphate soil disturbance

The average frequency this threat occurs at this habitat

- continuous

Consequence of this threat

- See **Survey Reference Sheet** for detailed instructions and definitions
- Select 1 number per row. 0 = no/minimal consequence, 4 = high consequence
- The scores for each scenario can be very different (e.g. 0, 2 and 4) if you are highly uncertain of the consequence or the same (e.g. 2, 2 and 2) if you are highly certain of the consequence

|  | **0** | **1** | **2** | **3** | **4** |
| --- | --- | --- | --- | --- | --- |
| Change in physical habitat structure (best-case scenario) | ( ) | ( ) | ( ) | ( ) | ( ) |
| Change in physical habitat structure (most-likely scenario) | ( ) | ( ) | ( ) | ( ) | ( ) |
| Change in physical habitat structure (worst-case scenario) | ( ) | ( ) | ( ) | ( ) | ( ) |
| Change in species composition and trophic structure (best-case scenario) | ( ) | ( ) | ( ) | ( ) | ( ) |
| Change in species composition and trophic structure (most-likely scenario) | ( ) | ( ) | ( ) | ( ) | ( ) |
| Change in species composition and trophic structure (worst-case scenario) | ( ) | ( ) | ( ) | ( ) | ( ) |

#### Comments:

#### Heavy metals

#### Point source and diffuse pollution from historical mining operations, stormwater, metal manufacture and power stations

The average frequency this threat occurs at this habitat

- continuous

Consequence of this threat

- See **Survey Reference Sheet** for detailed instructions and definitions
- Select 1 number per row. 0 = no/minimal consequence, 4 = high consequence
- The scores for each scenario can be very different (e.g. 0, 2 and 4) if you are highly uncertain of the consequence or the same (e.g. 2, 2 and 2) if you are highly certain of the consequence

|  | **0** | **1** | **2** | **3** | **4** |
| --- | --- | --- | --- | --- | --- |
| Change in physical habitat structure (best-case scenario) | ( ) | ( ) | ( ) | ( ) | ( ) |
| Change in physical habitat structure (most-likely scenario) | ( ) | ( ) | ( ) | ( ) | ( ) |
| Change in physical habitat structure (worst-case scenario) | ( ) | ( ) | ( ) | ( ) | ( ) |
| Change in species composition and trophic structure (best-case scenario) | ( ) | ( ) | ( ) | ( ) | ( ) |
| Change in species composition and trophic structure (most-likely scenario) | ( ) | ( ) | ( ) | ( ) | ( ) |
| Change in species composition and trophic structure (worst-case scenario) | ( ) | ( ) | ( ) | ( ) | ( ) |

#### Comments:

#### Disease and Pathogens

#### Outbreaks from naturally occurring and introduced species

The average frequency this threat occurs at this habitat

- rare (less than once a year)

Consequence of this threat

- See **Survey Reference Sheet** for detailed instructions and definitions
- Select 1 number per row. 0 = no/minimal consequence, 4 = high consequence
- The scores for each scenario can be very different (e.g. 0, 2 and 4) if you are highly uncertain of the consequence or the same (e.g. 2, 2 and 2) if you are highly certain of the consequence

|  | **0** | **1** | **2** | **3** | **4** |
| --- | --- | --- | --- | --- | --- |
| Change in physical habitat structure (best-case scenario) | ( ) | ( ) | ( ) | ( ) | ( ) |
| Change in physical habitat structure (most-likely scenario) | ( ) | ( ) | ( ) | ( ) | ( ) |
| Change in physical habitat structure (worst-case scenario) | ( ) | ( ) | ( ) | ( ) | ( ) |
| Change in species composition and trophic structure (best-case scenario) | ( ) | ( ) | ( ) | ( ) | ( ) |
| Change in species composition and trophic structure (most-likely scenario) | ( ) | ( ) | ( ) | ( ) | ( ) |
| Change in species composition and trophic structure (worst-case scenario) | ( ) | ( ) | ( ) | ( ) | ( ) |

#### Comments:

#### Marine debris

#### Includes general litter, fishing gear, aquaculture gear and illegal dumping

The average frequency this threat occurs at this habitat

- continuous

Consequence of this threat

- See **Survey Reference Sheet** for detailed instructions and definitions
- Select 1 number per row. 0 = no/minimal consequence, 4 = high consequence
- The scores for each scenario can be very different (e.g. 0, 2 and 4) if you are highly uncertain of the consequence or the same (e.g. 2, 2 and 2) if you are highly certain of the consequence

|  | **0** | **1** | **2** | **3** | **4** |
| --- | --- | --- | --- | --- | --- |
| Change in physical habitat structure (best-case scenario) | ( ) | ( ) | ( ) | ( ) | ( ) |
| Change in physical habitat structure (most-likely scenario) | ( ) | ( ) | ( ) | ( ) | ( ) |
| Change in physical habitat structure (worst-case scenario) | ( ) | ( ) | ( ) | ( ) | ( ) |
| Change in species composition and trophic structure (best-case scenario) | ( ) | ( ) | ( ) | ( ) | ( ) |
| Change in species composition and trophic structure (most-likely scenario) | ( ) | ( ) | ( ) | ( ) | ( ) |
| Change in species composition and trophic structure (worst-case scenario) | ( ) | ( ) | ( ) | ( ) | ( ) |

#### Comments:

#### Marine habitat modification: commercial harbours, ports

#### Sub-threats include alteration of currents/tides, sedimentation and coastal erosion

The average frequency this threat occurs at this habitat

- continuous

Consequence of this threat

- See **Survey Reference Sheet** for detailed instructions and definitions
- Select 1 number per row. 0 = no/minimal consequence, 4 = high consequence
- The scores for each scenario can be very different (e.g. 0, 2 and 4) if you are highly uncertain of the consequence or the same (e.g. 2, 2 and 2) if you are highly certain of the consequence

|  | **0** | **1** | **2** | **3** | **4** |
| --- | --- | --- | --- | --- | --- |
| Change in physical habitat structure (best-case scenario) | ( ) | ( ) | ( ) | ( ) | ( ) |
| Change in physical habitat structure (most-likely scenario) | ( ) | ( ) | ( ) | ( ) | ( ) |
| Change in physical habitat structure (worst-case scenario) | ( ) | ( ) | ( ) | ( ) | ( ) |
| Change in species composition and trophic structure (best-case scenario) | ( ) | ( ) | ( ) | ( ) | ( ) |
| Change in species composition and trophic structure (most-likely scenario) | ( ) | ( ) | ( ) | ( ) | ( ) |
| Change in species composition and trophic structure (worst-case scenario) | ( ) | ( ) | ( ) | ( ) | ( ) |

#### Comments:

#### Marine habitat modification: jetties, seawalls

#### Sub-threats include alteration of currents/tides, sedimentation and coastal erosion

The average frequency this threat occurs at this habitat

- continuous

Consequence of this threat

- See **Survey Reference Sheet** for detailed instructions and definitions
- Select 1 number per row. 0 = no/minimal consequence, 4 = high consequence
- The scores for each scenario can be very different (e.g. 0, 2 and 4) if you are highly uncertain of the consequence or the same (e.g. 2, 2 and 2) if you are highly certain of the consequence

|  | **0** | **1** | **2** | **3** | **4** |
| --- | --- | --- | --- | --- | --- |
| Change in physical habitat structure (best-case scenario) | ( ) | ( ) | ( ) | ( ) | ( ) |
| Change in physical habitat structure (most-likely scenario) | ( ) | ( ) | ( ) | ( ) | ( ) |
| Change in physical habitat structure (worst-case scenario) | ( ) | ( ) | ( ) | ( ) | ( ) |
| Change in species composition and trophic structure (best-case scenario) | ( ) | ( ) | ( ) | ( ) | ( ) |
| Change in species composition and trophic structure (most-likely scenario) | ( ) | ( ) | ( ) | ( ) | ( ) |
| Change in species composition and trophic structure (worst-case scenario) | ( ) | ( ) | ( ) | ( ) | ( ) |

#### Comments:

#### Marine habitat modification: marinas, boat ramps

#### Sub-threats include alteration of currents/tides, sedimentation and coastal erosion

The average frequency this threat occurs at this habitat

- continuous

Consequence of this threat

- See **Survey Reference Sheet** for detailed instructions and definitions
- Select 1 number per row. 0 = no/minimal consequence, 4 = high consequence
- The scores for each scenario can be very different (e.g. 0, 2 and 4) if you are highly uncertain of the consequence or the same (e.g. 2, 2 and 2) if you are highly certain of the consequence

|  | **0** | **1** | **2** | **3** | **4** |
| --- | --- | --- | --- | --- | --- |
| Change in physical habitat structure (best-case scenario) | ( ) | ( ) | ( ) | ( ) | ( ) |
| Change in physical habitat structure (most-likely scenario) | ( ) | ( ) | ( ) | ( ) | ( ) |
| Change in physical habitat structure (worst-case scenario) | ( ) | ( ) | ( ) | ( ) | ( ) |
| Change in species composition and trophic structure (best-case scenario) | ( ) | ( ) | ( ) | ( ) | ( ) |
| Change in species composition and trophic structure (most-likely scenario) | ( ) | ( ) | ( ) | ( ) | ( ) |
| Change in species composition and trophic structure (worst-case scenario) | ( ) | ( ) | ( ) | ( ) | ( ) |

#### Comments:

#### Marine habitat modification: dredging

#### Sub-threats include alteration of currents/tides and sediment re-suspension

The average frequency this threat occurs at this habitat

- rare (less than once a year)

Consequence of this threat

- See **Survey Reference Sheet** for detailed instructions and definitions
- Select 1 number per row. 0 = no/minimal consequence, 4 = high consequence
- The scores for each scenario can be very different (e.g. 0, 2 and 4) if you are highly uncertain of the consequence or the same (e.g. 2, 2 and 2) if you are highly certain of the consequence

|  | **0** | **1** | **2** | **3** | **4** |
| --- | --- | --- | --- | --- | --- |
| Change in physical habitat structure (best-case scenario) | ( ) | ( ) | ( ) | ( ) | ( ) |
| Change in physical habitat structure (most-likely scenario) | ( ) | ( ) | ( ) | ( ) | ( ) |
| Change in physical habitat structure (worst-case scenario) | ( ) | ( ) | ( ) | ( ) | ( ) |
| Change in species composition and trophic structure (best-case scenario) | ( ) | ( ) | ( ) | ( ) | ( ) |
| Change in species composition and trophic structure (most-likely scenario) | ( ) | ( ) | ( ) | ( ) | ( ) |
| Change in species composition and trophic structure (worst-case scenario) | ( ) | ( ) | ( ) | ( ) | ( ) |

#### Comments:

#### ****Nutrient discharge (point source)****

#### Mainly nitrogen and phosphorous

#### Point-source pollution from wastewater treatment plants, stormwater drains, steel manufacture, fish processors, power plants, land-based abalone farms, aquaculture (finfish) hatcheries, agricultural runoff via riverine/creek discharge and shark-cage berley

#### Does not include impacts from sea cage aquaculture

The average frequency this threat occurs at this habitat

- continuous

Consequence of this threat

- See **Survey Reference Sheet** for detailed instructions and definitions
- Select 1 number per row. 0 = no/minimal consequence, 4 = high consequence
- The scores for each scenario can be very different (e.g. 0, 2 and 4) if you are highly uncertain of the consequence or the same (e.g. 2, 2 and 2) if you are highly certain of the consequence

|  | **0** | **1** | **2** | **3** | **4** |
| --- | --- | --- | --- | --- | --- |
| Change in physical habitat structure (best-case scenario) | ( ) | ( ) | ( ) | ( ) | ( ) |
| Change in physical habitat structure (most-likely scenario) | ( ) | ( ) | ( ) | ( ) | ( ) |
| Change in physical habitat structure (worst-case scenario) | ( ) | ( ) | ( ) | ( ) | ( ) |
| Change in species composition and trophic structure (best-case scenario) | ( ) | ( ) | ( ) | ( ) | ( ) |
| Change in species composition and trophic structure (most-likely scenario) | ( ) | ( ) | ( ) | ( ) | ( ) |
| Change in species composition and trophic structure (worst-case scenario) | ( ) | ( ) | ( ) | ( ) | ( ) |

#### Comments:

#### Oil Spill, size: 100s of tonnes

#### The most-likely, worst-case oil spill for Spencer Gulf. For example, the 1992 ‘ERA’ spill: fuel source = ship’s fuel from ruptured fuel tank; fuel type = highly-persistent heavy fuel oil not easily broken down by chemical dispersants; spill size = 100s of tonnes.

The average frequency this threat occurs at this habitat

- rare (less than once a year)

Consequence of this threat

- See **Survey Reference Sheet** for detailed instructions and definitions
- Select 1 number per row. 0 = no/minimal consequence, 4 = high consequence
- The scores for each scenario can be very different (e.g. 0, 2 and 4) if you are highly uncertain of the consequence or the same (e.g. 2, 2 and 2) if you are highly certain of the consequence

|  | **0** | **1** | **2** | **3** | **4** |
| --- | --- | --- | --- | --- | --- |
| Change in physical habitat structure (best-case scenario) | ( ) | ( ) | ( ) | ( ) | ( ) |
| Change in physical habitat structure (most-likely scenario) | ( ) | ( ) | ( ) | ( ) | ( ) |
| Change in physical habitat structure (worst-case scenario) | ( ) | ( ) | ( ) | ( ) | ( ) |
| Change in species composition and trophic structure (best-case scenario) | ( ) | ( ) | ( ) | ( ) | ( ) |
| Change in species composition and trophic structure (most-likely scenario) | ( ) | ( ) | ( ) | ( ) | ( ) |
| Change in species composition and trophic structure (worst-case scenario) | ( ) | ( ) | ( ) | ( ) | ( ) |

#### Comments:

#### ****Fishing: pots****

#### blue crab and rock lobster, commercial and recreational

#### sub-threats include low level bycatch

The average frequency this threat occurs at this habitat

- 1 to 3 months/year

Consequence of this threat

- See **Survey Reference Sheet** for detailed instructions and definitions
- Select 1 number per row. 0 = no/minimal consequence, 4 = high consequence
- The scores for each scenario can be very different (e.g. 0, 2 and 4) if you are highly uncertain of the consequence or the same (e.g. 2, 2 and 2) if you are highly certain of the consequence

|  | **0** | **1** | **2** | **3** | **4** |
| --- | --- | --- | --- | --- | --- |
| Change in physical habitat structure (best-case scenario) | ( ) | ( ) | ( ) | ( ) | ( ) |
| Change in physical habitat structure (most-likely scenario) | ( ) | ( ) | ( ) | ( ) | ( ) |
| Change in physical habitat structure (worst-case scenario) | ( ) | ( ) | ( ) | ( ) | ( ) |
| Change in species composition and trophic structure (best-case scenario) | ( ) | ( ) | ( ) | ( ) | ( ) |
| Change in species composition and trophic structure (most-likely scenario) | ( ) | ( ) | ( ) | ( ) | ( ) |
| Change in species composition and trophic structure (worst-case scenario) | ( ) | ( ) | ( ) | ( ) | ( ) |

#### Comments:

#### ****Fishing: haul nets, gillnets****

#### scalefish, commercial and recreational

#### sub-threats include medium level bycatch

The average frequency this threat occurs at this habitat

- 1 to 3 months/year

Consequence of this threat

- See **Survey Reference Sheet** for detailed instructions and definitions
- Select 1 number per row. 0 = no/minimal consequence, 4 = high consequence
- The scores for each scenario can be very different (e.g. 0, 2 and 4) if you are highly uncertain of the consequence or the same (e.g. 2, 2 and 2) if you are highly certain of the consequence

|  | **0** | **1** | **2** | **3** | **4** |
| --- | --- | --- | --- | --- | --- |
| Change in physical habitat structure (best-case scenario) | ( ) | ( ) | ( ) | ( ) | ( ) |
| Change in physical habitat structure (most-likely scenario) | ( ) | ( ) | ( ) | ( ) | ( ) |
| Change in physical habitat structure (worst-case scenario) | ( ) | ( ) | ( ) | ( ) | ( ) |
| Change in species composition and trophic structure (best-case scenario) | ( ) | ( ) | ( ) | ( ) | ( ) |
| Change in species composition and trophic structure (most-likely scenario) | ( ) | ( ) | ( ) | ( ) | ( ) |
| Change in species composition and trophic structure (worst-case scenario) | ( ) | ( ) | ( ) | ( ) | ( ) |

#### Comments:

#### ****Fishing: handline, longline****

#### scalefish, commercial and recreational

#### sub-threats include low level bycatch

The average frequency this threat occurs at this habitat

- 3 to 9 months/year

Consequence of this threat

- See **Survey Reference Sheet** for detailed instructions and definitions
- Select 1 number per row. 0 = no/minimal consequence, 4 = high consequence
- The scores for each scenario can be very different (e.g. 0, 2 and 4) if you are highly uncertain of the consequence or the same (e.g. 2, 2 and 2) if you are highly certain of the consequence

|  | **0** | **1** | **2** | **3** | **4** |
| --- | --- | --- | --- | --- | --- |
| Change in physical habitat structure (best-case scenario) | ( ) | ( ) | ( ) | ( ) | ( ) |
| Change in physical habitat structure (most-likely scenario) | ( ) | ( ) | ( ) | ( ) | ( ) |
| Change in physical habitat structure (worst-case scenario) | ( ) | ( ) | ( ) | ( ) | ( ) |
| Change in species composition and trophic structure (best-case scenario) | ( ) | ( ) | ( ) | ( ) | ( ) |
| Change in species composition and trophic structure (most-likely scenario) | ( ) | ( ) | ( ) | ( ) | ( ) |
| Change in species composition and trophic structure (worst-case scenario) | ( ) | ( ) | ( ) | ( ) | ( ) |

#### Comments:

#### ****Fishing: demersal trawl****

#### western king prawns, commercial

#### sub-threats include high level bycatch and sediment re-suspension

The average frequency this threat occurs at this habitat

- 1 to 3 months/year

Consequence of this threat

- See **Survey Reference Sheet** for detailed instructions and definitions
- Select 1 number per row. 0 = no/minimal consequence, 4 = high consequence
- The scores for each scenario can be very different (e.g. 0, 2 and 4) if you are highly uncertain of the consequence or the same (e.g. 2, 2 and 2) if you are highly certain of the consequence

|  | **0** | **1** | **2** | **3** | **4** |
| --- | --- | --- | --- | --- | --- |
| Change in physical habitat structure (best-case scenario) | ( ) | ( ) | ( ) | ( ) | ( ) |
| Change in physical habitat structure (most-likely scenario) | ( ) | ( ) | ( ) | ( ) | ( ) |
| Change in physical habitat structure (worst-case scenario) | ( ) | ( ) | ( ) | ( ) | ( ) |
| Change in species composition and trophic structure (best-case scenario) | ( ) | ( ) | ( ) | ( ) | ( ) |
| Change in species composition and trophic structure (most-likely scenario) | ( ) | ( ) | ( ) | ( ) | ( ) |
| Change in species composition and trophic structure (worst-case scenario) | ( ) | ( ) | ( ) | ( ) | ( ) |

#### Comments:

**Fishing: purse seine**

- **sardines, commercial**
- **sub-threats include low level bycatch**

The average frequency this threat occurs at this habitat

- 3 to 9 months/year

Consequence of this threat

- See **Survey Reference Sheet** for detailed instructions and definitions
- Select 1 number per row. 0 = no/minimal consequence, 4 = high consequence
- The scores for each scenario can be very different (e.g. 0, 2 and 4) if you are highly uncertain of the consequence or the same (e.g. 2, 2 and 2) if you are highly certain of the consequence

|  | **0** | **1** | **2** | **3** | **4** |
| --- | --- | --- | --- | --- | --- |
| Change in physical habitat structure (best-case scenario) | ( ) | ( ) | ( ) | ( ) | ( ) |
| Change in physical habitat structure (most-likely scenario) | ( ) | ( ) | ( ) | ( ) | ( ) |
| Change in physical habitat structure (worst-case scenario) | ( ) | ( ) | ( ) | ( ) | ( ) |
| Change in species composition and trophic structure (best-case scenario) | ( ) | ( ) | ( ) | ( ) | ( ) |
| Change in species composition and trophic structure (most-likely scenario) | ( ) | ( ) | ( ) | ( ) | ( ) |
| Change in species composition and trophic structure (worst-case scenario) | ( ) | ( ) | ( ) | ( ) | ( ) |

#### Comments:

**Fishing: hand collection**

- **abalone, commercial and recreational**

The average frequency this threat occurs at this habitat

- 3 to 9 months/year

Consequence of this threat

- See **Survey Reference Sheet** for detailed instructions and definitions
- Select 1 number per row. 0 = no/minimal consequence, 4 = high consequence
- The scores for each scenario can be very different (e.g. 0, 2 and 4) if you are highly uncertain of the consequence or the same (e.g. 2, 2 and 2) if you are highly certain of the consequence

|  | **0** | **1** | **2** | **3** | **4** |
| --- | --- | --- | --- | --- | --- |
| Change in physical habitat structure (best-case scenario) | ( ) | ( ) | ( ) | ( ) | ( ) |
| Change in physical habitat structure (most-likely scenario) | ( ) | ( ) | ( ) | ( ) | ( ) |
| Change in physical habitat structure (worst-case scenario) | ( ) | ( ) | ( ) | ( ) | ( ) |
| Change in species composition and trophic structure (best-case scenario) | ( ) | ( ) | ( ) | ( ) | ( ) |
| Change in species composition and trophic structure (most-likely scenario) | ( ) | ( ) | ( ) | ( ) | ( ) |
| Change in species composition and trophic structure (worst-case scenario) | ( ) | ( ) | ( ) | ( ) | ( ) |

#### Comments:

#### ****Fishing: illegal****

The average frequency this threat occurs at this habitat

- 1 to 30 days/year

Consequence of this threat

- See **Survey Reference Sheet** for detailed instructions and definitions
- Select 1 number per row. 0 = no/minimal consequence, 4 = high consequence
- The scores for each scenario can be very different (e.g. 0, 2 and 4) if you are highly uncertain of the consequence or the same (e.g. 2, 2 and 2) if you are highly certain of the consequence

|  | **0** | **1** | **2** | **3** | **4** |
| --- | --- | --- | --- | --- | --- |
| Change in physical habitat structure (best-case scenario) | ( ) | ( ) | ( ) | ( ) | ( ) |
| Change in physical habitat structure (most-likely scenario) | ( ) | ( ) | ( ) | ( ) | ( ) |
| Change in physical habitat structure (worst-case scenario) | ( ) | ( ) | ( ) | ( ) | ( ) |
| Change in species composition and trophic structure (best-case scenario) | ( ) | ( ) | ( ) | ( ) | ( ) |
| Change in species composition and trophic structure (most-likely scenario) | ( ) | ( ) | ( ) | ( ) | ( ) |
| Change in species composition and trophic structure (worst-case scenario) | ( ) | ( ) | ( ) | ( ) | ( ) |

#### Comments:

#### Invasive species: benthic filter-feeders

#### Key species include European fanworm, Pacific oyster and pearl oyster

The average frequency this threat occurs at this habitat

- continuous

Consequence of this threat

- See **Survey Reference Sheet** for detailed instructions and definitions
- Select 1 number per row. 0 = no/minimal consequence, 4 = high consequence
- The scores for each scenario can be very different (e.g. 0, 2 and 4) if you are highly uncertain of the consequence or the same (e.g. 2, 2 and 2) if you are highly certain of the consequence

|  | **0** | **1** | **2** | **3** | **4** |
| --- | --- | --- | --- | --- | --- |
| Change in physical habitat structure (best-case scenario) | ( ) | ( ) | ( ) | ( ) | ( ) |
| Change in physical habitat structure (most-likely scenario) | ( ) | ( ) | ( ) | ( ) | ( ) |
| Change in physical habitat structure (worst-case scenario) | ( ) | ( ) | ( ) | ( ) | ( ) |
| Change in species composition and trophic structure (best-case scenario) | ( ) | ( ) | ( ) | ( ) | ( ) |
| Change in species composition and trophic structure (most-likely scenario) | ( ) | ( ) | ( ) | ( ) | ( ) |
| Change in species composition and trophic structure (worst-case scenario) | ( ) | ( ) | ( ) | ( ) | ( ) |

#### Comments:

#### Invasive species: predators, parasites

#### Key species include crabs, goby fish and shell-boring worms

The average frequency this threat occurs at this habitat

- continuous

Consequence of this threat

- See **Survey Reference Sheet** for detailed instructions and definitions
- Select 1 number per row. 0 = no/minimal consequence, 4 = high consequence
- The scores for each scenario can be very different (e.g. 0, 2 and 4) if you are highly uncertain of the consequence or the same (e.g. 2, 2 and 2) if you are
- highly certain of the consequence

|  | **0** | **1** | **2** | **3** | **4** |
| --- | --- | --- | --- | --- | --- |
| Change in physical habitat structure (best-case scenario) | ( ) | ( ) | ( ) | ( ) | ( ) |
| Change in physical habitat structure (most-likely scenario) | ( ) | ( ) | ( ) | ( ) | ( ) |
| Change in physical habitat structure (worst-case scenario) | ( ) | ( ) | ( ) | ( ) | ( ) |
| Change in species composition and trophic structure (best-case scenario) | ( ) | ( ) | ( ) | ( ) | ( ) |
| Change in species composition and trophic structure (most-likely scenario) | ( ) | ( ) | ( ) | ( ) | ( ) |
| Change in species composition and trophic structure (worst-case scenario) | ( ) | ( ) | ( ) | ( ) | ( ) |

#### Comments:

#### Invasive species: encrusting, fouling species

#### Key species include ascidians, hydroids and macroalgae

The average frequency this threat occurs at this habitat

- 3 to 9 months/year

Consequence of this threat

- See **Survey Reference Sheet** for detailed instructions and definitions
- Select 1 number per row. 0 = no/minimal consequence, 4 = high consequence
- The scores for each scenario can be very different (e.g. 0, 2 and 4) if you are highly uncertain of the consequence or the same (e.g. 2, 2 and 2) if you are highly certain of the consequence

|  | **0** | **1** | **2** | **3** | **4** |
| --- | --- | --- | --- | --- | --- |
| Change in physical habitat structure (best-case scenario) | ( ) | ( ) | ( ) | ( ) | ( ) |
| Change in physical habitat structure (most-likely scenario) | ( ) | ( ) | ( ) | ( ) | ( ) |
| Change in physical habitat structure (worst-case scenario) | ( ) | ( ) | ( ) | ( ) | ( ) |
| Change in species composition and trophic structure (best-case scenario) | ( ) | ( ) | ( ) | ( ) | ( ) |
| Change in species composition and trophic structure (most-likely scenario) | ( ) | ( ) | ( ) | ( ) | ( ) |
| Change in species composition and trophic structure (worst-case scenario) | ( ) | ( ) | ( ) | ( ) | ( ) |

#### Comments:

#### Harmful algal blooms

#### Outbreaks from naturally occurring and introduced species

The average frequency this threat occurs at this habitat

- rare (less than once a year)

Consequence of this threat

- See **Survey Reference Sheet** for detailed instructions and definitions
- Select 1 number per row. 0 = no/minimal consequence, 4 = high consequence
- The scores for each scenario can be very different (e.g. 0, 2 and 4) if you are highly uncertain of the consequence or the same (e.g. 2, 2 and 2) if you are highly certain of the consequence

|  | **0** | **1** | **2** | **3** | **4** |
| --- | --- | --- | --- | --- | --- |
| Change in physical habitat structure (best-case scenario) | ( ) | ( ) | ( ) | ( ) | ( ) |
| Change in physical habitat structure (most-likely scenario) | ( ) | ( ) | ( ) | ( ) | ( ) |
| Change in physical habitat structure (worst-case scenario) | ( ) | ( ) | ( ) | ( ) | ( ) |
| Change in species composition and trophic structure (best-case scenario) | ( ) | ( ) | ( ) | ( ) | ( ) |
| Change in species composition and trophic structure (most-likely scenario) | ( ) | ( ) | ( ) | ( ) | ( ) |
| Change in species composition and trophic structure (worst-case scenario) | ( ) | ( ) | ( ) | ( ) | ( ) |

#### Comments:

#### Sediment runoff and dust

#### From land clearance and coastal habitat modification

The average frequency this threat occurs at this habitat

- continuous

Consequence of this threat

- See **Survey Reference Sheet** for detailed instructions and definitions
- Select 1 number per row. 0 = no/minimal consequence, 4 = high consequence
- The scores for each scenario can be very different (e.g. 0, 2 and 4) if you are highly uncertain of the consequence or the same (e.g. 2, 2 and 2) if you are highly certain of the consequence

|  | **0** | **1** | **2** | **3** | **4** |
| --- | --- | --- | --- | --- | --- |
| Change in physical habitat structure (best-case scenario) | ( ) | ( ) | ( ) | ( ) | ( ) |
| Change in physical habitat structure (most-likely scenario) | ( ) | ( ) | ( ) | ( ) | ( ) |
| Change in physical habitat structure (worst-case scenario) | ( ) | ( ) | ( ) | ( ) | ( ) |
| Change in species composition and trophic structure (best-case scenario) | ( ) | ( ) | ( ) | ( ) | ( ) |
| Change in species composition and trophic structure (most-likely scenario) | ( ) | ( ) | ( ) | ( ) | ( ) |
| Change in species composition and trophic structure (worst-case scenario) | ( ) | ( ) | ( ) | ( ) | ( ) |

#### Comments:

### ****Shipping****

### Sub-threats include sediment re-suspension, anchor damage, waste discharge, noise and antifoulants.

### Does not include threats related to invasive species or oil spills.

#### The average frequency this threat occurs at this habitat

#### near-continuous to continuous (see below)

Consequence of this threat

- See **Survey Reference Sheet** for detailed instructions and definitions
- Select 1 number per row. 0 = no/minimal consequence, 4 = high consequence
- The scores for each scenario can be very different (e.g. 0, 2 and 4) if you are highly uncertain of the consequence or the same (e.g. 2, 2 and 2) if you are highly certain of the consequence

#### PART A: ****Low**** shipping intensity (< 100 ships crossing habitat per year)

|  | **0** | **1** | **2** | **3** | **4** |
| --- | --- | --- | --- | --- | --- |
| Change in physical habitat structure (best-case scenario) | ( ) | ( ) | ( ) | ( ) | ( ) |
| Change in physical habitat structure (most-likely scenario) | ( ) | ( ) | ( ) | ( ) | ( ) |
| Change in physical habitat structure (worst-case scenario) | ( ) | ( ) | ( ) | ( ) | ( ) |
| Change in species composition and trophic structure (best-case scenario) | ( ) | ( ) | ( ) | ( ) | ( ) |
| Change in species composition and trophic structure (most-likely scenario) | ( ) | ( ) | ( ) | ( ) | ( ) |
| Change in species composition and trophic structure (worst-case scenario) | ( ) | ( ) | ( ) | ( ) | ( ) |

#### Comments:

#### PART B: ****Medium**** shipping intensity (100 to 300 ships crossing habitat per year)

|  | **0** | **1** | **2** | **3** | **4** |
| --- | --- | --- | --- | --- | --- |
| Change in physical habitat structure (best-case scenario) | ( ) | ( ) | ( ) | ( ) | ( ) |
| Change in physical habitat structure (most-likely scenario) | ( ) | ( ) | ( ) | ( ) | ( ) |
| Change in physical habitat structure (worst-case scenario) | ( ) | ( ) | ( ) | ( ) | ( ) |
| Change in species composition and trophic structure (best-case scenario) | ( ) | ( ) | ( ) | ( ) | ( ) |
| Change in species composition and trophic structure (most-likely scenario) | ( ) | ( ) | ( ) | ( ) | ( ) |
| Change in species composition and trophic structure (worst-case scenario) | ( ) | ( ) | ( ) | ( ) | ( ) |

#### Comments:

#### PART C: ****High**** shipping intensity (300 to 600 ships crossing habitat per year)

|  | **0** | **1** | **2** | **3** | **4** |
| --- | --- | --- | --- | --- | --- |
| Change in physical habitat structure (best-case scenario) | ( ) | ( ) | ( ) | ( ) | ( ) |
| Change in physical habitat structure (most-likely scenario) | ( ) | ( ) | ( ) | ( ) | ( ) |
| Change in physical habitat structure (worst-case scenario) | ( ) | ( ) | ( ) | ( ) | ( ) |
| Change in species composition and trophic structure (best-case scenario) | ( ) | ( ) | ( ) | ( ) | ( ) |
| Change in species composition and trophic structure (most-likely scenario) | ( ) | ( ) | ( ) | ( ) | ( ) |
| Change in species composition and trophic structure (worst-case scenario) | ( ) | ( ) | ( ) | ( ) | ( ) |

#### Comments:

#### Thermal pollution

#### Point-source from power plants and steel manufacture

The average frequency this threat occurs at this habitat

- continuous

Consequence of this threat

- See **Survey Reference Sheet** for detailed instructions and definitions
- Select 1 number per row. 0 = no/minimal consequence, 4 = high consequence
- The scores for each scenario can be very different (e.g. 0, 2 and 4) if you are highly uncertain of the consequence or the same (e.g. 2, 2 and 2) if you are highly certain of the consequence

|  | **0** | **1** | **2** | **3** | **4** |
| --- | --- | --- | --- | --- | --- |
| Change in physical habitat structure (best-case scenario) | ( ) | ( ) | ( ) | ( ) | ( ) |
| Change in physical habitat structure (most-likely scenario) | ( ) | ( ) | ( ) | ( ) | ( ) |
| Change in physical habitat structure (worst-case scenario) | ( ) | ( ) | ( ) | ( ) | ( ) |
| Change in species composition and trophic structure (best-case scenario) | ( ) | ( ) | ( ) | ( ) | ( ) |
| Change in species composition and trophic structure (most-likely scenario) | ( ) | ( ) | ( ) | ( ) | ( ) |
| Change in species composition and trophic structure (worst-case scenario) | ( ) | ( ) | ( ) | ( ) | ( ) |

#### Comments:

#### Part IV: Assessing natural recovery potential of your habitat to threat disturbance

#### ****Recovery time of habitat**** Please indicate the average number of months or years required for your habitat to return to its ‘near-normal’ state once it has been completely destroyed by a threat (e.g. a threat that would receive a consequence score of 4). Recovery time is also based on the assumption that threats do not re-occur during the recovery period. For biotic habitats, recovery time may relate to age-at-maturity of keystone species.

( ) unsure of recovery time

( ) less than 1 month

( ) 1 to 12 months

( ) 1 to 5 years

( ) 5 to 10 years

( ) 10 to 50 years

( ) more than 50 years

#### ****Frequency of natural disturbance**** Please indicate the average frequency your habitat is exposed to significant natural disturbance.

( ) unsure of frequency

( ) daily to weekly

( ) several times a year

( ) once a year

( ) every 1 to 5 years

( ) more than every 5 years

#### ****Habitat patchiness**** Please indicate the current level of habitat patchiness in Spencer Gulf. Patchiness is defined as complete breaks in habitat that would restrict the re-establishment of propagules in a disturbed area.

( ) unsure of patchiness

( ) continuous habitat

( ) moderately patchy habitat

( ) highly patchy habitat

#### Part V: Assessing synergistic interactions among threats

#### Please indicate if any of the threats you assessed are *likely* to interact synergistically with another threat in your chosen habitat, so that the sum of their combined impact is greater than the sum of their individual impacts.

#### If you are unsure about synergistic interactions among threats, indicate here and move to the next question

[ ] I am unsure about synergistic interactions

You can select up to 3 interactions per threat (i.e. 1 interaction per column). You DO NOT need to fill in every box.

*A large table of drop down lists is displayed here*

**Part VI: Feedback**

#### Would you like a pdf summary of your survey answers?

( ) Yes

( ) No

#### In resulting publications, would you like to be acknowledged (name and affiliation identified) as participating in the survey?

( ) Yes

( ) No

#### Would you like to be kept informed of resulting publications that will arise from this project?

( ) Yes

( ) No

### Any other comments or queries?

____________________________________________

____________________________________________

____________________________________________

____________________________________________

**SURVEY REFERENCE SHEET**

**Part III: Assessing the ecological consequence of each threat to your habitat**

In Part III, the bulk of the survey, you will be asked to assess the ecological consequence of individual threats to your habitat for a given level of threat exposure.

You will be asked to provide a score from 0 (no/minimal consequence) to 4 (high consequence) for two Consequence Indicators (**see next page for detailed definitions** of each Indicator):

1. Change in physical habitat structure
2. Change in species composition and trophic structure

**Level of threat exposure**

To help you assess the consequence of a threat, the extent to which your habitat is exposed to each threat on a **temporal** basis (i.e. frequency) is provided on the survey. For example:

The average frequency that “Fishing (pots)” occurs on “Algal Forest & Rock Reef” is:

- 3 to 9 months/year

Note: **Spatial** exposure (i.e. the percent area that a habitat in Spencer Gulf is exposed to a threat) will be incorporated into the risk assessment post survey.

**Scenarios and Uncertainty**

To incorporate **level of uncertainty** associated with your estimated consequence, there will be three sets of scores for each Consequence Indicator:

- best-case scenario (lowest or equal score), plausible best-case scenario given your level of uncertainty
- most-likely scenario (middle or equal score), your best estimate or guess
- worst-case scenario (highest or equal score), plausible worst-case scenario given your level of uncertainty

The three sets of scores can be very different if you are highly uncertain of the consequence or the same is you are highly certain of the consequence. For example:

| Scenario | Highly **uncertain**  of consequence | Highly **certain**  of consequence |
| --- | --- | --- |
| Best-case | 0 | 2 |
| Most-likely | 2 | 2 |
| Worst-case | 4 | 2 |

**Consequence Indicators (detailed definitions)**

*1) Change in physical habitat structure*

Key Question: What is the % change in the physical properties of the habitat from exposure to the threat?

Physical properties include abiotic components (substrate, water column, geochemical attributes) and habitat-forming biota. Includes both short and long-term changes.

Score criteria:

0 = no, minimal or positive change

1 = 1–30% change in habitat structure

2 = 30-60% change in habitat structure

3 = 60-90% change in habitat structure

4 = 90-100 % change in habitat structure (near-complete loss of native habitat)

Hypothetical examples:

*Heatwave threat - seagrass habitat:* A heatwave may result in a 70% loss in seagrass cover and score 3 (i.e. 60 – 90% change in habitat structure).

*Shipping threat – pelagic habitat:* Shipping may only alter pelagic habitats to a very small extent (ie. changes to biogeochemical components from waste discharge and antifoulants) and score 1 (i.e. 1–30% change in habitat structure).

*2) Change in species composition and trophic structure*

Key Question: What is the change in the species composition and trophic structure of the habitat from exposure to the threat?

Includes both short and long-term changes.

Score criteria:

0 = no, positive or minimal change

1 = significantly alters the abundance of 1 species

2 = significantly alters the abundance of several species or 1 trophic level

3 = significantly alters the abundance of numerous species or several trophic levels

4 = significantly alters the abundance of all species in the habitat (ie entire food web)

Hypothetical examples:

*Marine habitat modification threat - soft intertidal habitat:* This small-scale discreet threat may significantly alter the abundance of several species (i.e. infauna) and score 1.

*Ocean acidification threat - soft bottom habitat:* This ubiquitous and continuous threat may alter the abundance of all species living in subtidal soft bottom habitats and score 4.
